# Reconstructing the Stem Leydig Cell Niche via the Testicular Extracellular Matrix for the Treatment of Testicular Leydig Cell Dysfunction

**DOI:** 10.1002/advs.202410808

**Published:** 2024-11-18

**Authors:** Ani Chi, Chao Yang, Jie Liu, Zhichen Zhai, Xuetao Shi

**Affiliations:** ^1^ National Engineering Research Centre for Tissue Restoration and Reconstruction Key Laboratory of Biomedical Engineering of Guangdong Province South China University of Technology Guangzhou 510640 P. R. China; ^2^ Key Laboratory of Biomedical Engineering of Guangdong Province South China University of Technology Guangzhou 510006 P. R. China; ^3^ School of Materials Science and Engineering South China University of Technology Guangzhou 510640 P. R. China

**Keywords:** aging, extracellular matrix, Leydig cell, stem Leydig cell

## Abstract

Therapies involving the use of stem Leydig cells (SLCs), as testicular mesenchymal stromal cells, have shown great promise in the treatment of Leydig cell (LC) dysfunction in aging males. However, the outcomes of these therapies are not satisfactory. In this study, it is demonstrated that the aging microenvironment of the testicular interstitium impairs the function of SLCs, leading to poor regeneration of LCs and, consequently, inefficient functional restoration. The study develops a decellularized testicular extracellular matrix (dTECM) hydrogel from young pigs and evaluates its safety and feasibility as a supportive niche for the expansion and differentiation of SLCs. dTECM hydrogel facilitates the steroidogenic differentiation of SLCs into LCs, the primary producers of testosterone. The combination of SLCs with a dTECM hydrogel leads to a significant and sustained increase in testosterone levels, which promotes the restoration of spermatogenesis and fertility in an LC‐deficient and aged mouse model. Mechanistically, collagen 1 within the dTECM is identified as a key factor contributing to these effects. Notably, symptoms associated with testosterone deficiency syndrome are significantly alleviated in aged mice. These findings may aid the design of therapeutic interventions for patients with testosterone deficiency in the clinic.

## Graphical abstract

Graphical abstract showing the mechanism by which dTECM‐SLC‐based therapy ameliorates testicular LC dysfunction. Local injection of dTECM‐SLC into the testicular interstitium. dTECM creates a favorable microenvironment for SLC proliferation and differentiation. dTECM‐SLC effectively increases testosterone levels and reshapes the local immune microenvironment in the testicular interstitium. After treatment, symptoms associated with testosterone deficiency are ultimately alleviated, as evidenced by increased muscle mass, alleviation of osteoporosis, loss of white fat volume, and improved cognitive function.

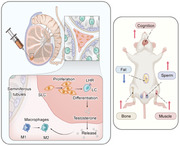

## 1 Introduction

As testicular multipotent stromal cells, stem Leydig cells (SLCs) act as a reserve progenitor population that is essential for testis development, homeostasis, and regeneration. SLCs are capable of proliferating and differentiating into Leydig cells (LCs),^[^ ^1^ ^]^ which are responsible for the production of the majority of testosterone.^[^ ^2^ ^]^ However, the aging of SLCs is associated with a decline in regenerative capacity and multilineage differentiation potential, contributing to the development of testosterone deficiency in aging males.^[^ ^3^ ^]^ Our previous study reported that exogenous SLC transplantation therapies have shown great promise in the treatment of LC dysfunction in aged mice^[^ ^4^ ^]^ and nonhuman primate models,^[^ ^5^ ^]^ overcoming many of the shortcomings of testosterone therapy in the clinic. However, these therapies restore testosterone levels for a relatively short period. Effective bioengineering strategies for improving therapeutic outcomes after transplantation are still lacking.

Stem cell function is critical for tissue homeostasis and dependent on interactions with the surrounding microenvironment, which is known as the “stem cell niche”.^[^ ^6^, ^7^, ^8^ ^]^. Recent studies have suggested that an aging niche contributes to a decrease in stem cell function.^[^ ^8^, ^9^, ^10^ ^]^ SLCs located in the testicular interstitium are surrounded by Sertoli cells, LCs, peritubular myoid cells, and macrophages, which interact with SLCs via the release of soluble factors and direct cell‒cell communication, providing cues for SLC activation, proliferation, and differentiation.^[^ ^11^ ^]^ A previous study revealed dramatic upregulation of inflammation‐induced genes in these somatic cells in aging testes.^[^ ^12^ ^]^ Our previous study also has revealed that exosome proteins in the aging testicular interstitium are enriched in the functions “regulation of response to oxidative stress”, “cellular senescence”, and “activation of immune response”.^[^ ^13^ ^]^ All of these factors suggest that the stem cell niche changes during aging in a manner that is detrimental to the function of SLCs. Notably, increasing evidence suggests that a “young” microenvironment can restore the functions of aged stem cell.^[^ ^14^, ^15^ ^]^For example, aged hematopoietic stem cells can be rejuvenated in a young bone marrow niche.^[^ ^16^ ^]^ Inspired by this, we hypothesize that the restoration of a young niche for transplanted SLCs may be a promising method for achieving cell regeneration in aging testes.

As a crucial noncellular component of the SLC niche, the testicular extracellular matrix (ECM), which is distributed throughout the seminiferous basement membrane and testicular interstitium, contains many proteins required for spermatogenesis.^[^ ^17^ ^]^ The ECM not only provides structural support but also regulates niche biochemical signals crucial for tissue maintenance and regeneration.^[^ ^18^ ^]^ ECM remodeling is intricately coordinated with stem cell niche activity, ensuring the orderly replenishment of cell lineages within organs.^[^ ^19^ ^]^ However, during aging, the levels of ECM constituents undergo significant changes, which are accompanied by cellular senescence,^[^ ^20^, ^21^ ^]^ contributing to the failure of stem cell maintenance.^[^ ^22^, ^23^ ^]^ Decellularized extracellular matrix (dECM) can be used as a substitute for the cell niche for the generation of engineered tissues.^[^ ^24^ ^]^ Tissue‐specific ECM‐based hydrogels obtained via decellularization have shown strong potential for supporting the regeneration of various tissues and organs, including the heart,^[^ ^25^ ^]^ liver,^[^ ^26^ ^]^ and skin.^[^ ^27^ ^]^ Decellularized testicular extracellular matrix (dTECM) has been used as a scaffold for 3D culture of testicular cells for the study of spermatogenesis in vitro,^[^ ^28^ ^]^ suggesting the potential of dTECM to reconstruct the native cellular microenvironment. However, the effect of dTECM on the SLC niche is largely unexplored.

Here, we demonstrated that the aging microenvironment within the testicular interstitium impaired the function of SLCs. To address this, we developed a dTECM hydrogel that promoted the proliferation and differentiation of SLCs into LCs. This approach effectively and sustainably increased testosterone levels, thereby restoring spermatogenesis and fertility in LC‐deficient and aged mice. Notably, this process ameliorated aging‐related symptoms associated with testosterone deficiency, as evidenced by increased muscle mass, alleviation of osteoporosis, loss of white fat volume and improved cognitive function. Collectively, our findings reveal that the use of young dTECM represents a novel therapeutic approach to support the regenerative activity of SLCs and address LC dysfunction in aging males.

## 2 Results

### 2.1 The Aging Niche Substantially Compromises SLC Function

**Figure 1:**
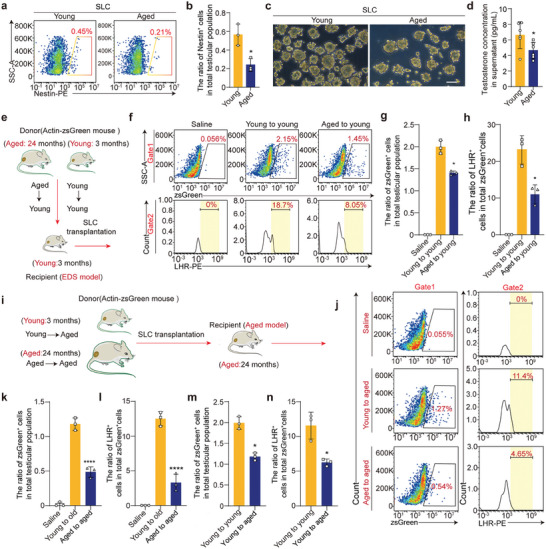
Characteristics of young and aged SLCs transplanted into the testicular interstitium of young and aged mice. a) Flow cytometry analysis of the percentage of SLCs in the testes of mice from different age groups. b) Quantification of the percentage of SLCs in the testes of mice from different age groups. The data are presented as the means ± SDs. n = 3 biological replicates for each group. Unpaired two‐tailed Student's *t*‐test was used. c) Bright field image of young and aged SLC clones. Scale bar, 100 µm. d) Quantitative analysis of testosterone production in vitro. The data are presented as the means ± SDs. n = 3 biological replicates for each group. Unpaired two‐tailed Student's *t*‐test was used. e) Experimental strategy for the evaluation of SLC survival and differentiation in EDS‐treated mice. f) Flow cytometry analysis of the percentage of SLCs^ZsGreen^ and LHR^+^ZsGreen^+^ cells among total testicular cells in the young‐to‐young and aged‐to‐young groups on day 28. g,h) Quantitative analysis of the ratio of SLCs^ZsGreen^ and LHR^+^ZsGreen^+^ cells to total testicular cells in the young‐to‐young and aged‐to‐young groups on day 28. The data are presented as the means ± SDs. n = 3 biological replicates for each group. One‐way ANOVA was used. i) Experimental strategy for the evaluation of SLC survival and differentiation in the testes of aged mice. j) Flow cytometry was used to measure the percentage of SLCs^ZsGreen^ and LHR^+^ZsGreen^+^ cells relative to total testicular cells in the young‐to‐ aged and aged‐to‐ aged groups on day 28. k,l) Quantitative analysis of the ratio of SLCs^ZsGreen^ and LHR^+^ZsGreen^+^ cells to total testicular cells in the young‐to‐ aged and aged‐to‐ aged groups on day 28. The data are presented as the means ± SDs. n = 3 biological replicates for each group. One‐way ANOVA was used. m,n) Quantitative analysis of the numbers of SLCs^ZsGreen^, ZsGreen^+^LHR^+^ cells, and total testicular cells in the young‐to‐young and young‐to‐ aged groups on day 28. The data are presented as the means ± SDs. n = 3 biological replicates for each group. An unpaired two‐tailed Student's *t*‐test was used. *p < 0.05, ****p < 0.0001.

To investigate how aging impacts SLCs, we first compared the functions of these cells in young and aged mice. We observed a significant reduction in the abundance of SLCs, identified as Nestin^+^ cells, in aged mice relative to the total number of testicular cells (**Figure** **1**A,B; Figure S1A, Supporting Information). We harvested SLCs from mouse testes and induced them to differentiate in vitro. SLCs from aged mice exhibited reduced clonogenic efficiency (Figure 1C) and diminished potential for differentiation into LCs, leading to decreased testosterone production in vivo (Figure 1D). Loss of differentiation capacity is considered a key indicator of stem cell aging.^[^ ^29^ ^]^ Consistent with previous reports, these results suggest that the proliferation and differentiation capacity of SLCs are impaired in aged mice.

Stem cells play crucial roles in tissue homeostasis and are regulated by the niche microenvironment.^[^ ^7^ ^]^ We next compared the effects of young and aged niches on SLCs by using both young and aged SLCs to investigate how the niche influences SLC function. First, we transplanted young and aged SLCs into 3‐month‐old mice treated with ethane dimethanesulfonate (EDS) to construct a model of LC deficiency and assessed the ability of the SLCs to regenerate damaged tissues in the young niche. Three‐month‐old mice were intraperitoneally injected with EDS solution (160 mg/kg) (Figure S1B, Supporting Information). After 4 days, immunofluorescence staining revealed a significant reduction in the number of LCs, and the serum testosterone levels were markedly decreased (Figure S1C, D, Supporting Information). Then, equal numbers of young and aged SLCs^zsGreen^ were transplanted into the testicular interstitium (Figure 1E; Figure S1E Supporting Information). On day 28, flow cytometry revealed that the percentage of young SLCs^zsGreen^ surviving in the testes of EDS‐treated mice was significantly greater than the percentage of aged SLCs^zsGreen^ surviving in the testes of EDS‐treated mice (Figure 1F,G). Importantly, the proportion of LHR^+^zsGreen^+^ cells (LHR is a marker of LCs) in the young group was significantly greater than that in the aged group (Figure 1H). These results suggest that the steroidogenic lineage differentiation capacity of aged SLCs is decreased, which highlights that compared with young SLCs, aged SLCs exhibit age‐associated cellular deficits. We then transplanted young or aged SLCs into 24‐month‐old male mice to examine their ability to regenerate damaged tissues in the aged niche. An equal number of young or aged SLCs^zsGreen^ were transplanted into the testes of aged mice (Figure 1I). On day 28, the percentage of surviving young SLCs^zsGreen^ was significantly greater than that of surviving aged SLCs^zsGreen^ (Figure 1J,K), and the percentage of young LHR^+^zsGreen^+^ cells was also significantly greater than that of aged LHR^+^zsGreen^+^ cells (Figure 1L). Notably, in addition to the above results, we determined that the percentage of surviving SLCs^zsGreen^, as well as the percentage of LHR^+^zsGreen^+^ cells (Figure 1M,N), was significantly greater in the testes of young mice than in those of aged mice, suggesting that the exposure of young SLCs to the aged testicular interstitium impaired rejuvenation, whereas the transplantation of aged SLCs into the young testicular interstitium improved treatment outcomes, suggesting that the aging microenvironment is hostile to SLCs.

Collectively, these results indicate that the aged niche impairs the survival of SLCs and decreases their steroidogenic differentiation capacity, whereas the young niche supports more favorable outcomes of SLC transplantation.

### 2.2 Reconstructing the SLC Niche via Young dTECM

**Figure 2:**
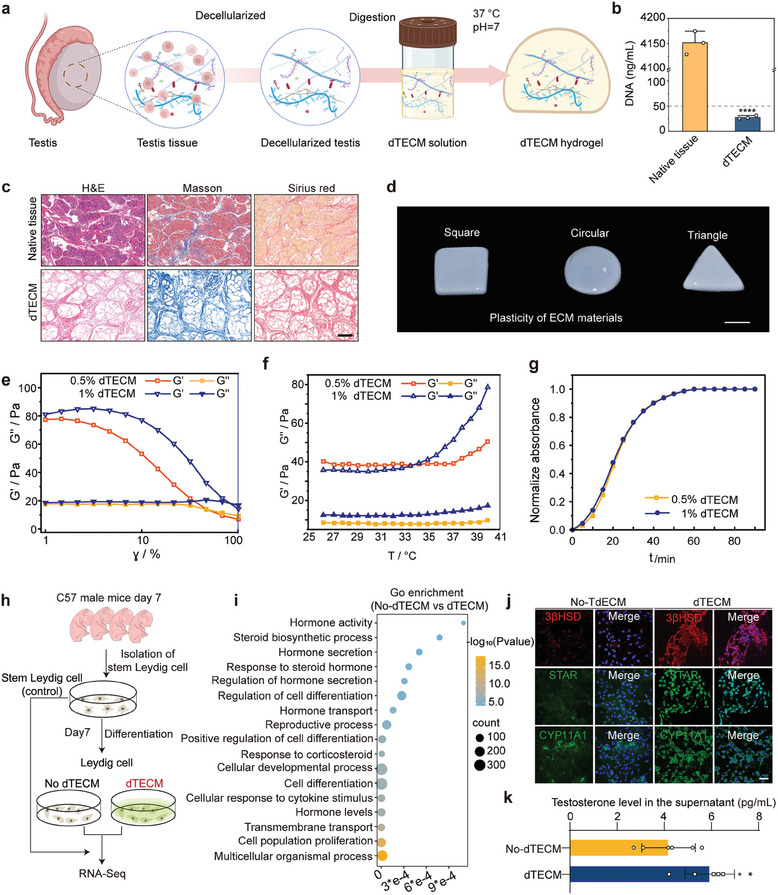
Young dTECM hydrogels promote SLC proliferation and differentiation in vitro. a) Schematic illustration of the process used to prepare the dTECM hydrogel. b) Quantification of DNA levels in native testis tissue and dTECM. The data are presented as the means ± SDs. n = 3 biological replicates for each group. An unpaired two‐tailed Student's *t*‐test was used. c) H&E, MT, and Sirius red staining indicated that the cells had been removed and that collagen was preserved after decellularization. Scale bar, 125 µm. d) Photograph of the obtained dTECM gels of different shapes. Scale bar, 1 cm. e) Rheology analysis of the dTECM hydrogels in the dynamic frequency sweep experiment. f) Dynamic modulus of the dTECM hydrogels as a function of temperature. g) Spectrophotometry was used to assess the turbidity of the samples during gelation. h) Schematic illustration of the culture and differentiation of SLCs with or without dTECM. i) Representative GO terms associated with the upregulated genes. A hypergeometric test was used for statistical analysis. j) At seven days after differentiation, immunofluorescence staining revealed that the cells in the no‐dTECM and dTECM groups expressed LC lineage‐specific markers, including 3βHSD, LHR, and CYP11A1. Scale bar, 50 µm. k) Quantitative analysis of testosterone production in the cell supernatants of the no‐dTECM and dTECM groups in vitro. The data are presented as the means ± SDs. n = 6 biological replicates for each group. An unpaired two‐tailed Student's t test was used. *p < 0.05, ****p < 0.0001.

Niche remodeling strategies have been extensively employed to functionalize niches for organ regeneration.^[^ ^30^ ^]^ The SLC niche comprises somatic cells, soluble factors, and testis tissue‐specific ECM. Specifically, the ECM is synthesized and secreted by various cells and contains a complex and spatially organized mixture of macromolecules that can mimic all aspects of the natural tissue microenvironment, providing a stable microenvironment for stem cell growth, proliferation, and differentiation.^[^ ^31^ ^]^ Therefore, we propose the integration of somatic cell types (SLCs) with testis‐specific dECM as a strategy to increase the efficacy of transplantation therapy. We reconstructed dTECM from young pigs, which are readily available in large quantities and have long been the preferred choice for dTECM preparation. Porcine testis tissue was harvested and dissected into small fragments, followed by decellularization, lyophilization, gamma irradiation, digestion, and neutralization (**Figure** **2**A; Figure S2A,B, Supporting Information). Histological analysis with hematoxylin and eosin (H&E), Masson's trichrome (MT), and Sirius red staining revealed that most of the cells had been removed, while the DNA content in the dTECM was 27.81 ng/mL, which is equivalent to the 0.669% DNA content observed in native testis tissue (Figure 2B,C; Figure S2C, Supporting Information). Taken together, these results indicate that most of the cellular components were removed successfully by the dTECM preparation process.

Minimally invasive strategies are necessary for delivering agents to the narrow testicular interstitium; therefore, we designed dTECM with flexible mechanical properties and temperature‐sensitive gel formation properties to accommodate testis‐specific structures and avoid causing mechanical damage. We determined suitable conditions for the formation of dTECM hydrogels that could conform to any 3D shape. The appropriate ECM concentration, pH, and temperature were evaluated. The ECM digestion solution, which had a pH of 7.4 and a concentration of 5 or 10 mg/mL, could form a hydrogel at 37 °C. By casting the neutralized dTECM pregel into a mold, ECM hydrogels with different shapes, including hemispherical, rectangular and triangular shapes, were successfully prepared (Figure 2D). Representative scanning electron microscopy (SEM) images revealed that the surface microstructure of the dTECM hydrogels was highly porous and had a compact microarchitecture (Figure S2D, Supporting Information). Then, we assessed the rheological characteristics of the reconstructed dTECM via amplitude sweep rheology. The hydrogels were incubated at 37 °C on a rheometer for testing, and the results revealed that G' was greater than G'', indicating that the hydrogel molding process was successful. As the sweep temperature approached and remained at 37 °C, a sudden increase in the storage modulus was observed for the dTECM pregels, indicating the presence of collagenous fibers and the ability of the pregels to undergo thermal crosslinking and transition from the pregel state to a stabilized hydrogel state (Figure 2E,F; Figure S2E,F, Supporting Information). The turbidity test results revealed that the hydrogels could be completely cured within 50 min (Figure 2G; Figure S2G,H, Supporting Information).

We next examined the effect of dTECM on SLCs in vitro. SLCs were isolated from the tests of 7‐day‐old mice (Figure 2H; Figure S3A, Supporting Information), as SLCs are most abundant at this age. After 7 days of in vitro culture, the isolated primary SLCs proliferated and formed floating clonal spheres (Figure S3B,C, Supporting Information). Immunostaining revealed that the SLCs highly expressed Nestin and PDGFR‐α but were negative for the LC markers LHR and 3β‐HSD (Figure S3D, Supporting Information), indicating that the SLCs maintained an undifferentiated state. The SLCs were cultured with dTECM gels at concentrations of 5 mg/mL or 10 mg/mL for 14 days. Live/dead staining revealed no hydrogel‐induced cytotoxicity, and the SLCs exhibited a greater proliferation rate in the 5 mg/mL dTECM gels than in the 10 mg/mL dTECM gels (Figure S3E, Supporting Information). A CCK‐8 assay confirmed these results (Figure S3F, Supporting Information). Thus, 5 mg/mL gels were used for subsequent experiments. These results demonstrate that dTECM gels have good biocompatibility and biological functionality and can support the adhesion and proliferation of SLCs.

SLCs can differentiate into LCs with a normal testosterone production capacity, which is key for the successful treatment of testosterone deficiency via stem cell replacement therapy.^[^ ^32^ ^]^ Therefore, we assessed whether dTECM affects the differentiation of SLCs. SLCs were harvested and induced to differentiate in vitro. After 7 days of culture, RNA sequencing (RNA‐seq) analysis of the global transcriptome of SLCs (control), SLCs differentiated without dTECM (no‐dTECM), and SLCs differentiated with dTECM (dTECM) was conducted (Figure 2H). We detected clear differences in the transcriptome profiles of the SLCs in the control, no‐dTECM, and dTECM groups (Figure S4A, Supporting Information). There were 2398 downregulated and 1462 upregulated differentially expressed genes (DEGs) between the control and no‐dTECM groups (Figure S4B,C, Supporting Information). GO and KEGG analyses of the upregulated genes revealed that they were enriched in processes such as the “cell surface receptor signaling pathway” and “focal adhesion” (Figure S4D,E, Supporting Information). Gene set enrichment analysis (GSEA) revealed significantly increased expression of genes related to “steroid hormone biosynthesis” (Figure S4F,G, Supporting Information), suggesting that SLCs transitioned from a stem cell state to a differentiated state in this differentiation culture system. There were 694 downregulated and 1127 upregulated DEGs between the no‐dTECM group and the dTECM group (Figures S4B and S5A,B, Supporting Information). Gene Ontology (GO) analysis and Kyoto Encyclopedia of Genes and Genomes (KEGG) analysis of the upregulated genes in the TdEM group revealed that they were enriched in processes such as “steroid biosynthetic process”, “regulation of cell differentiation”, “cell population proliferation” and “ECM‐receptor interaction” (Figure 2I; Figure S5C, Supporting Information), suggesting that dTECM plays an important role in promoting SLC proliferation and differentiation. Specifically, the expression of genes related to steroid biosynthetic synthesis, such as *Hsd11b1*, *Cyp11b1* and *Cyp11a1*, was significantly elevated (Figure S5D, Supporting Information). Consistent with these results, immunofluorescence staining revealed that the SLCs in the dTECM group produced more mature LCs (Figure 2J). Testosterone levels were greater in the dTECM group than in the no‐dTECM group (Figure 2K). Taken together, these data confirm that dTECM is able to maintain cell viability, promote cell proliferation, and promote SLC differentiation.

Finally, we assessed the biocompatibility and safety of dTECM in mice by locally injecting 20 µL of dTECM into the testicular interstitium. The mice were weighed on days 0, 7, 14, 21, and 28 after injection. There were no significant differences in body weight between the dTECM and saline groups (Figure S6A,B, Supporting Information). Blood biochemistry analysis revealed no toxicity at the tested dose or within the tested time frame (Figure S6C, Supporting Information). There were no significant changes in vital organs (Figure S6D, Supporting Information). Taken together, these results demonstrate that dTECM adapt to the testicular tissue structure without causing cytotoxicity.

### 2.3 dTECM is Conducive to the Proliferation and Differentiation of SLCs in an LC‐Deficient Mouse Model

**Figure 3:**
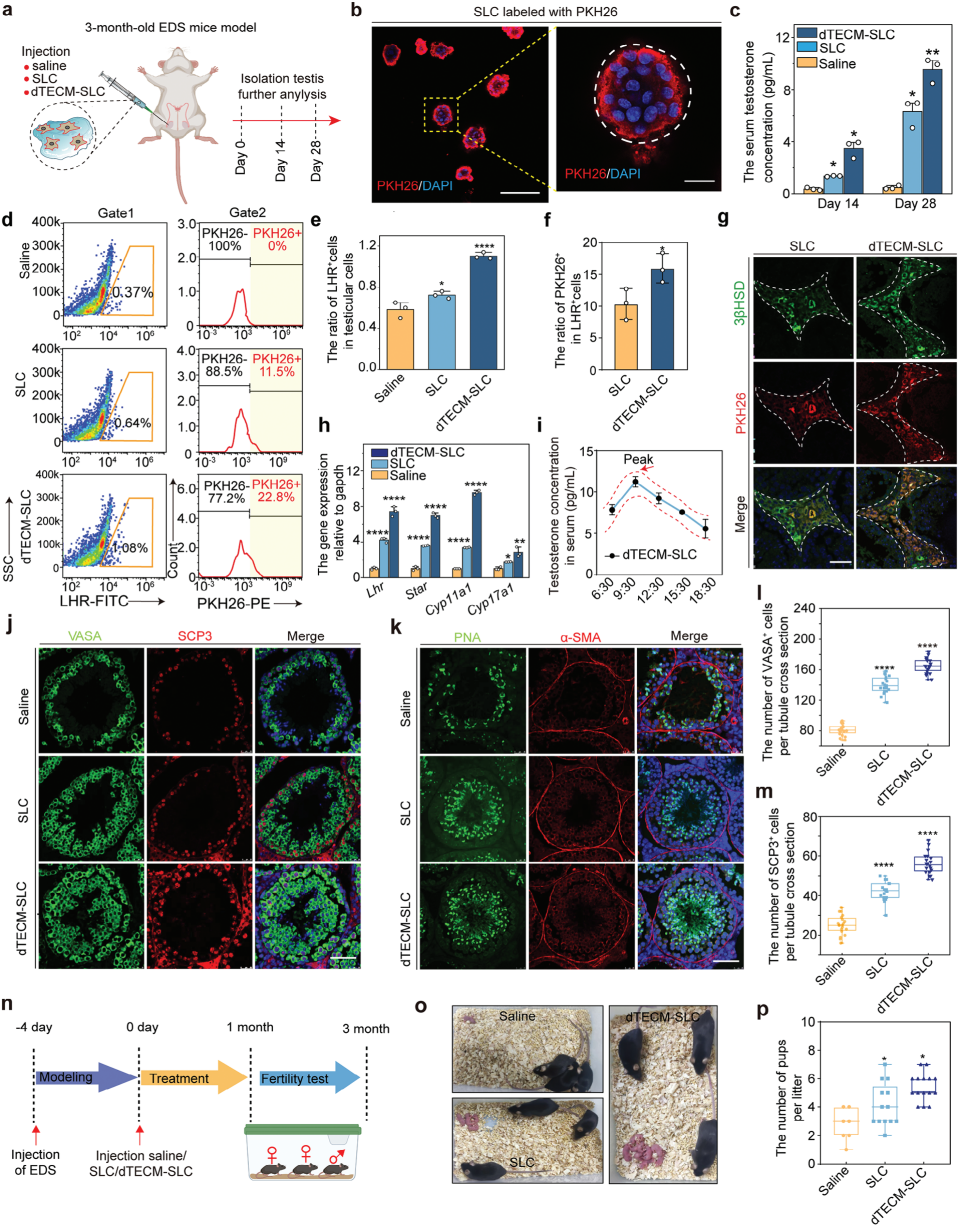
dTECM‐SLC treatment promotes the recovery of testosterone levels and spermatogenesis in EDS‐treated mice. a) Schematic illustration of dTECM‐SLC injection into the testicular interstitium and analysis of treatment effects in a mouse model of EDS‐induced LC deficiency. b) Representative images of SLCs labeled with PKH26. Scale bar, 100 µm; scale bar in the enlarged image, 25 µm. c) Serum testosterone concentrations were measured on days 14 and 28 in each group. n = 3 biological replicates for each group. The data are presented as the means ± SDs, and one‐way ANOVA was used. d) Flow cytometry was used to determine the percentages of LHR^+^ and LHR^+^ PKH26^+^ cells relative to total testicular cells in the saline, SLC and dTECM‐SLC groups on day 28. e,f) Quantitative analysis of the percentages of LHR^+^ and LHR^+^ PKH26^+^ cells relative to total testicular cells in the saline, SLC and dTECM‐SLC groups on day 28. n = 3 biological replicates for each group. The data are presented as the means ± SDs, and one‐way ANOVA was used. g) Immunofluorescence staining showing PKH26^+^ cells (red) stained with 3βHSD (green) in the testicular interstitium at 28 days. Scale bar, 50 µm. h) RT‒PCR analysis of the relative mRNA expression of LC markers in the testes of mice from the saline, SLC and dTECM‐SLC treatment groups. n = 3 biological replicates for each group. The data are presented as the means ± SDs, and one‐way ANOVA was used. i) Consecutive measurements of serum testosterone levels revealed that they exhibited a circadian rhythm in the dTECM‐SLC treatment groups. The red arrows indicate peak values. The red dotted line represents the trend. n = 3 biological replicates. The data are presented as the means ± SDs. j,k) Immunostaining of VASA (green), SCP3 (red), α‐SMA (red), and PNA (green) at 28 days. Scale bar, 50 µm. l,m) Quantitative analysis of the number of VASA^+^ and SCP3^+^ cells in seminiferous tubules per section in the saline, SLC and dTECM‐SLC groups. n = 3 biological replicates for each group. The data are presented as the means ± SDs, and one‐way ANOVA was used. n) Timeline of the experimental steps for mating assessment. o) Photograph of offspring in the saline, SLC and dTECM‐SLC groups. p) Quantitative analysis of the number of pups per litter in the saline, SLC and dTECM‐SLC groups. n = 6 biological replicates for each group. The data are presented as the means ± SDs, and one‐way ANOVA was used. *p < 0.05, **p < 0.01, ****p < 0.0001.

We further investigated the effect of dTECM on the differentiation of SLCs into LCs in vivo. SLCs were labeled with PKH26 and locally injected into the testicular interstitium (**Figure** **3**A,B). In vivo fluorescence imaging revealed a gradual decrease in the proportion of SLCs from day 1 to day 7, and the percentage of dTECM‐SLCs was significantly greater than that of SLCs (Figure S7A, Supporting Information). Moreover, immunofluorescence analysis revealed a further decrease in the number of SLCs from days 14–28, while the number of SLCs in the dTECM group was significantly greater on day 28 than on day 14 (Figure S7B,C, Supporting Information). These findings suggested that dTECM promoted the proliferation of SLCs in vivo. Notably, the serum testosterone concentration in the dTECM‐SLC group was significantly greater than that in the SLC group at days 14 and 28 (Figure 3C). Flow cytometry analysis revealed that the proportion of PKH26^+^ cells in the dTECM‐SLC group was significantly greater than that in the SLC group (Figure 3D,E; Figures S8A and S9A, Supporting Information). Among the PKH26^+^ cell population, the proportion of LHR^+^ PKH26^+^ cells was greater in the dTECM‐SLC group than in the SLC group (Figure 3F; Figure S9B, Supporting Information), indicating that dTECM promoted SLC differentiation into LCs. Immunofluorescence staining revealed that more 3βHSD^+^PKH26^+^ cells were found in the testicular interstitium in the dTECM‐SLC group than in the other groups (Figure 3G). The gene expression of LC markers, such as *Cyp11a1*, *Lhr*, 3*βHsd*, and *Star*, was consistently greater in the dTECM‐SLC group than in the SLC group or saline group (Figure 3H; Figure S9C, Supporting Information). Importantly, testosterone secretion exhibited a circadian rhythm, suggesting that the circulating concentration of testosterone was regulated by the hypothalamic‒pituitary‒gonadal axis in mice in the dTECM‐SLC group (Figure 3I). Taken together, these results indicate that dTECM can effectively promote the differentiation of SLCs and increase testosterone levels in vivo.

Spermatogenic dysfunction is frequently linked to impaired LC function.^[^ ^33^ ^]^ We next assessed the ability of transplanted dTECM‐SLCs to maintain spermatogenesis. Histopathological imaging revealed a significant increase in the thickness of the seminiferous tubules and germ cells in the seminiferous epithelium (Figure S10A, Supporting Information). Moreover, the proportions of cells expressing VASA (a germ cell marker) and synaptonemal complex protein 3 (SCP3, a specific marker of spermatogenesis) and PNA^+^ (a marker of sperm or haploid spermatids) cells were significantly greater in the dTECM‐SLC group than in the SLC group (Figure 3M; Figure S10B, Supporting Information). Accordingly, the sperm density significantly increased (Figure S10C, Supporting Information). Next, we conducted experiments to evaluate the impact of dTECM‐SLC treatment on mouse fertility (Figure 3O). Each male mouse was mated with female mice at a ratio of 1:2 within 2 months. The number of pups per litter was greater in the dTECM‐SLC group than in the SLC group (Figure 3P,Q). On the basis of the above results, we conclude that dTECM can effectively improve the outcomes of SLC treatment, increasing spermatogenesis and fertility in LC‐deficient male mice.

### 2.4 dTECM‐SLC Treatment Promotes Testosterone Recovery and Ameliorates Chronic Inflammation in the Testicular Interstitium of Aged Mice

**Figure 4:**
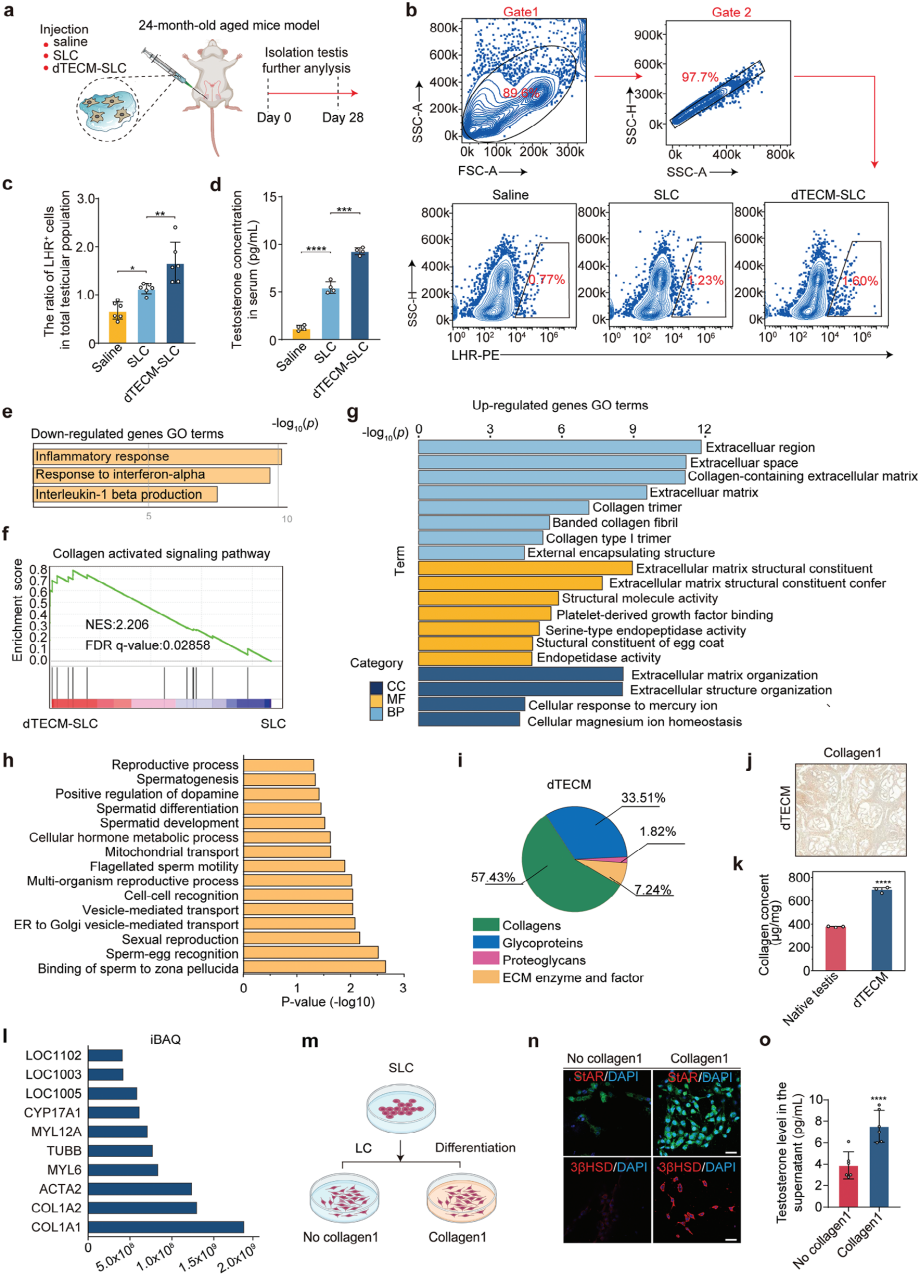
dTECM improves the therapeutic outcomes of SLC through collagen 1. a) Schematic illustration of dTECM‐SLC injection into the testicular interstitium and analysis of the treatment effects in an aged mouse model. b) Flow cytometry was used to determine the percentages of LHR^+^ cells relative to total testicular cells in the saline, SLC and dTECM‐SLC groups on day 28. c,d) The percentage of LHR^+^ cells and the serum testosterone concentration were measured on day 28. n = 3 biological replicates for each group. The data are presented as the means ± SDs, and one‐way ANOVA was used. e) Representative GO terms for the genes downregulated in the dTECM‐SLC group. A hypergeometric test was used for statistical analysis. f) GSEA showing the collagen‐activating pathways with increased activity in the dTECM‐SLC group. g) Representative GO terms for the genes upregulated in the dTECM‐SLC group. A hypergeometric test was used for statistical analysis. h) Biological processes in which the proteins expressed in dTECM were enriched according to GO enrichment analyses. i) Proteomic analyses with the matrisome database (MatrisomeDB) were performed to determine the percentages of matrisome proteins expressed in dTECM. j) Immunohistochemical staining of collagen 1. Scale bar, 125 µm. k) Quantification of collagen levels in native testis tissue and dTECM. The data are presented as the means ± SDs. n = 3 biological replicates for each group. An unpaired two‐tailed Student's *t*‐test was used. l) Top ten most abundant proteins in dTECM according to proteomics analysis.m) Schematic illustration of the culture and differentiation of SLCs with or without collagen 1. n) At seven days after differentiation, immunofluorescence staining revealed that the cells in the no‐ collagen 1 and collagen 1 groups expressed LC lineage‐specific markers, including 3βHSD and STAR. Scale bar, 10 µm. o) Quantitative analysis of testosterone production in the cell supernatants of the no‐ collagen 1 and collagen 1 groups in vitro. The data are presented as the means ± SDs. n = 6 biological replicates for each group. An unpaired two‐tailed Student's *t*‐test was used. *p < 0.05, **p < 0.01, **p < 0.001, ****p < 0.0001.

Next, we evaluated the therapeutic effects of dTECM‐SLCs in an aged mouse model. Equal volumes of saline, SLCs, or dTECM‐SLCs were injected into the testes of 24‐month‐old mice (**Figure** **4**A). On day 28, there were more LHR^+^ cells in the dTECM‐SLC group than in the SLC group (Figure 4B,C), and the serum testosterone level in the dTECM‐SLC group was significantly greater than that in the SLC group (Figure 4D). These results confirmed that dTECM‐SLC treatment promoted testosterone recovery after transplantation into the testicular interstitium in aged mice. To comprehensively elucidate the changes in the testicular interstitial microenvironment, we compared the transcriptomes of testicular interstitial cells of aged mice between the SLC and dTECM‐SLC groups via RNA‐seq. There were marked variations in the transcriptome profiles (Figure S11A–C, Supporting Information). Notably, the downregulated genes were enriched in the “chemokine signaling pathway” and “cytokine‒cytokine receptor interaction” pathways (Figure S11D, Supporting Information). GSEA revealed significantly decreased expression of genes related to “positive regulation of interferon‐β” and “production and response to interferon‐α” (Figure S11E,F, Supporting Information). In addition, we observed a greater abundance of CD206^+^ anti‐inflammatory M2 macrophages in the dTECM‐SLC group than in the SLC group (Figure S11G,H, Supporting Information), suggesting a shift toward an inflammatory phenotype. These results further confirm that dTECM‐SLC treatment can significantly alleviate chronic inflammation related to testicular aging, which may have been attributable to elevated testosterone levels, as testosterone is known to inhibit the production of inflammatory cytokines.^[^ ^34^ ^]^ Moreover, SA‐β‐gal staining analysis indicated a reduction in senescence within the testicular interstitium (Figure S11I, Supporting Information). All of the above results indicate that dTECM‐SLC treatment can significantly alleviate testicular aging‐related chronic inflammation within the testicular interstitium.

### 2.5 dTECM Promotes the Therapeutic Effects of SLC through Collagen 1

Homeostasis of the microenvironment in the interstitium is essential for testis function. However, how dTECM promotes the maintenance of microenvironment homeostasis in the interstitium by SLCs is unclear. To answer this question, we further analyzed the RNA‐seq data described above. Surprisingly, GSEA revealed significantly increased expression of genes related to the “collagen activated signaling pathway”. DEGs upregulated in the dTECM‐SLC group were enriched in processes such as “extracellular matrix structural constituent”, “collagen type I trimer”, and “collagen‐containing extracellular matrix” (Figure 4F,G). These results strongly suggest that collagen may be associated with dTECM, promoting the therapeutic effects of SLC. To verify our hypothesis, we further analyzed the proteins expressed in dTECM via mass spectrometry (MS). More than 574 proteins were identified. GO and KEGG analyses revealed that the testis tissue‐associated proteins expressed in dTECM were involved in “cellular hormone metabolic processes”, “spermatid development” and “spermatogenesis” (Figure 4H; Figures S12A,B and S13A, Supporting Information). These proteins can be categorized into four groups: collagens, proteoglycans, glycoproteins, and ECM enzymes and factors, of which there were relatively fewer (Figure 4I). Collagen was the most abundant component of dTECM, with the collagen content in dTECM being 183.63% of that in native testis tissue (Figure 4J,K). Notably, collagen 1 was the most abundant protein in dTECM (Figure 4L). Considering that collagen 1 is a well‐known ECM molecule that is conducive to stem cell proliferation and differentiation,^[^ ^35^, ^36^ ^]^ we focused on validating the function of collagen 1 in SLCs (Figure 4M). SLCs were seeded on collagen I and induced to differentiate in vitro. After 7 days of culture, there were more 3βHSD‐ or StAR‐positive cells in the collagen 1 group than in the control group (Figure 4N), and the testosterone level was also significantly increased (Figure 4O). Collectively, these data demonstrate the important role of collagen 1 in the promotion of SLC proliferation and differentiation, which promotes the recovery of testosterone levels in mice.

### 2.6 dTECM‐SLC Treatment Prevents a Decrease in Fertility in Aged Mice

**Figure 5:**
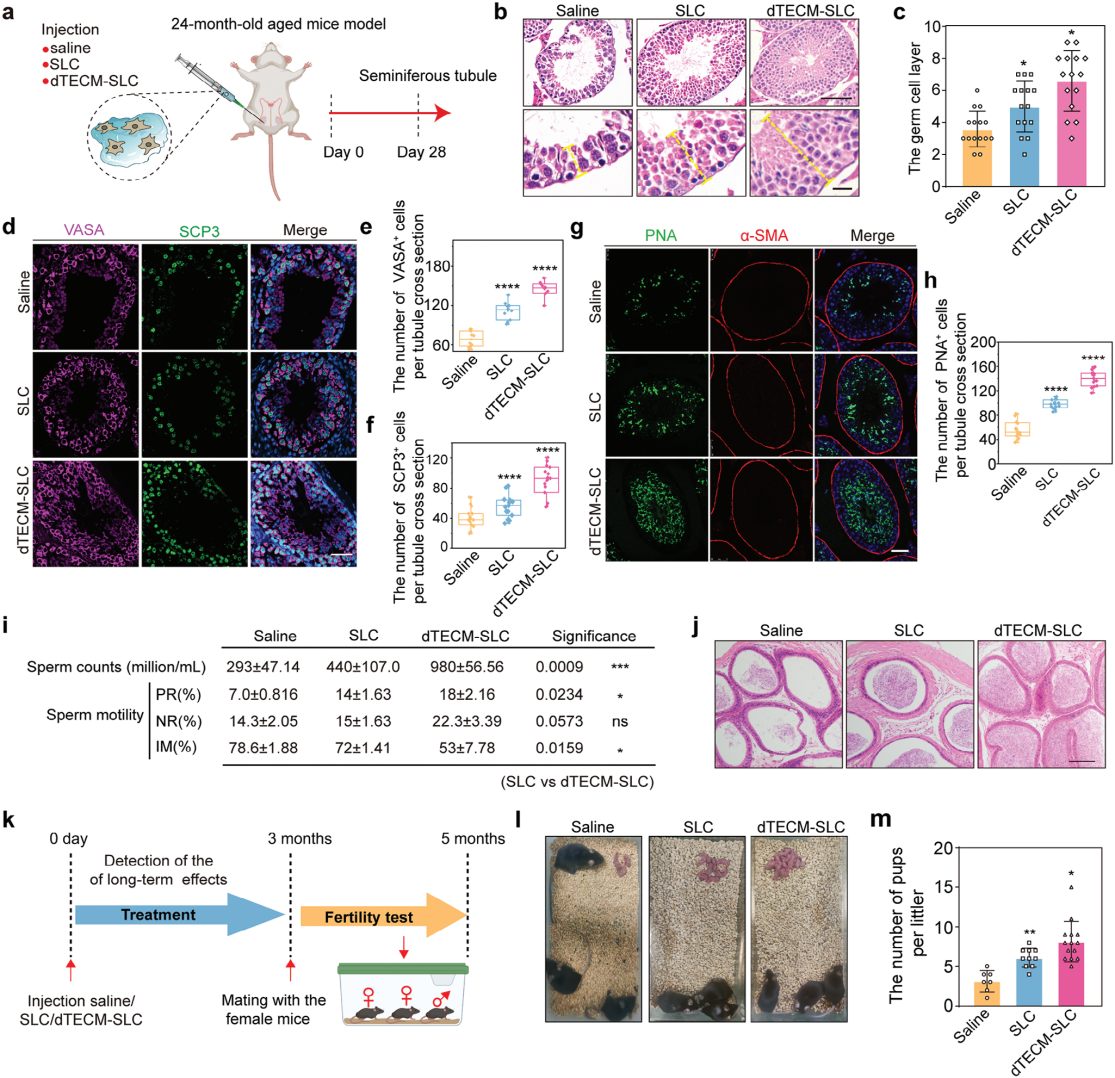
dTECM promote spermatogenesis and improve fertility in aged mice. a) Schematic illustration of dTECM‐SLC injection into the testicular interstitium and analysis of treatment effects in an aged mouse model. b) H&E staining of testis samples obtained from aged mice in the saline, SLC and dTECM‐SLC groups on day 28. The dotted line shows the thickness of the spermatogenic epithelium. Scale bar, 50 µm; scale bar in the enlarged image, 50 µm. c) Quantification of the number of germ cell layers. n = 6 biological replicates for each group. The data are presented as the means ± SDs, and one‐way ANOVA was used. d) Immunostaining of the germ cell markers VASA (purple) and SCP3 (green) at 28 days. Scale bar, 50 µm. e,f) Quantitative analysis of the average number of VASA^+^ and SCP3^+^ cells in seminiferous tubules per section in the saline, SLC and dTECM‐SLC groups. n = 6 biological replicates for each group. The data are presented as the means ± SDs, and one‐way ANOVA was used. g) Immunostaining of α‐SMA (red) and PNA (green) at 28 days. Scale bar, 50 µm. h) Quantitative analysis of the number of PNA^+^ cells in seminiferous tubules per section in the saline, SLC and dTECM‐SLC groups. n = 6 biological replicates for each group. The data are presented as the means ± SDs, and one‐way ANOVA was used. i) Sperm counts and sperm motility levels in the saline, SLC and dTECM‐SLC groups. PR: progressive motility, NP: nonprogressive motility, IM: immotility. n = 3 biological replicates for each group. The data are presented as the means ± SDs. One‐way ANOVA was used. j) Histological analysis of cauda epididymides. Scale bars, 50 µm. k) Timeline of the experimental steps for mating assessment. l) Photograph of offspring in the saline, SLC and dTECM‐SLC groups. m) Quantitative analysis of the number of pups per litter in the saline, SLC and dTECM‐SLC groups. n = 6 biological replicates for each group. The data are presented as the means ± SDs, and one‐way ANOVA was used.*p < 0.05, **p < 0.01, ***p < 0.001, ****p < 0.0001. ns, not significant.

Testosterone is indispensable for the maturation of male germ cells and the production of sperm.^[^^]^ Thus, we further investigated whether dTECM‐SLC treatment could effectively restore spermatogenesis. After 28 days of treatment, the thickness of the seminiferous tubules and the number of germ cells in the seminiferous epithelium were significantly greater in the dTECM‐SLC group than in the SLC group (**Figure** **5**A–C). In addition, dTECM‐SLC effectively restored the expression levels of germ cell markers, including VASA and SCP3, which were severely decreased in aged male mice, and there were significantly more PNA^+^ cells in the dTECM‐SLC group than in the SLC group (Figure 5D–H). Importantly, semen analysis indicated that sperm counts and motility were notably greater in the dTECM‐SLC group than in the SLC and saline groups (Figure 5I,J). These results suggest that dTECM‐SLC promotes spermatogenesis in aged mice.

To further investigate the long‐term effects on mouse fertility, 24‐month‐old male mice were randomly injected with saline, SLCs, or dTECM‐SLC. After 3 months, male mice were mated with 3‐month‐old female mice at a ratio of 1:2 to explore the effects of dTECM‐SLC treatment on their offspring (Figure 5K). The results revealed that mice the dTECM‐SLC group had more pups and more litters than mice in the SLC and saline groups did (Figure 5L,M). Taken together, these results indicate that dTECM‐SLC treatment can effectively promote spermatogenesis and increase fertility in aged mice.

### 2.7 dTECM‐SLC Treatment Alleviates Testosterone Deficiency‐Related Symptoms in Aged Mice

**Figure 6:**
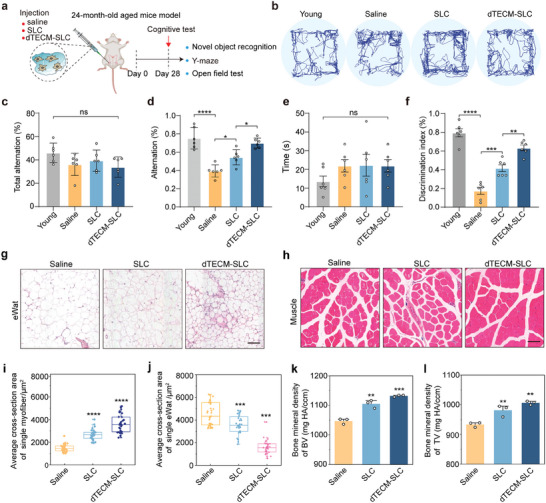
dTECM promotes improvements in cognitive ability, muscle atrophy, fat accumulation, and bone density in aged mice. a) Schematic illustration of the cognitive tests. b) Representative images of movement paths in the NOR test. c,d) Effects of treatment on spontaneous alternation behavior in the Y maze test. n = 6 biological replicates for each group. The data are presented as the means ± SDs, and one‐way ANOVA was used. e) The total time spent exploring the novel object in the NOR test. n = 6 biological replicates for each group. The data are presented as the means ± SDs, and one‐way ANOVA was used. f) Effects of treatment on the discrimination index in the NOR test. n = 6 biological replicates for each group. The data are presented as the means ± SDs, and one‐way ANOVA was used. g,h) Representative images of muscle and epididymal fat cross‐sections from saline‐, SLC‐ and dTECM‐SLC‐treated mice. Scale bars, 25 µm. i,j) Quantitative analysis of the average muscle and epididymal fat cross‐sectional areas. n = 6 biological replicates for each group. The data are presented as the means ± SDs, and one‐way ANOVA was used. k,l) Quantitative analysis of the femurs of the mice. BMD, bone mineral density. BV, bone volume; TV, tissue volume. n = 3 biological replicates for each group. The data are presented as the means ± SDs, and one‐way ANOVA was used. *p < 0.05, **p < 0.01, ***p < 0.001, ****p < 0.0001. ns, not significant.

Given that age‐related deficiencies in testosterone levels are associated with physiological changes, such as cognitive decline, osteoporosis, obesity, and decreased muscle mass,^[^ ^37^ ^]^ we further examined whether dTECM‐SLC transplantation is sufficient to alleviate the physiological impairments associated with aging. To assess the ability of dTECM to improve working memory and cognition, all the mice were subjected to the Y maze test (**Figure** **6**A,B; Figure S14A, Supporting Information). Compared with young mice, aged mice exhibited a marked decrease in the spontaneous alternation rate in the saline group; this reduction in spontaneous alternation behavior was attenuated by treatment with SLC, and dTECM‐SLC exhibited the most favorable treatment outcomes (Figure 6C,D; Figure S14B,C, Supporting Information). The discrimination time and discrimination index in the novel object recognition (NOR) test are classic indices used to assess visual learning and memory. We found that the mice treated with dTECM‐SLC showed a greater preference for the novel object than did the mice in the SLC group (Figure 6E,F; Figure S14D,E, Supporting Information). The results of the open field test revealed that there was no obvious difference in athletic ability among the groups (Figure S14F,G, Supporting Information). Taken together, these results suggest that dTECM‐SLC treatment is sufficient to alleviate age‐related cognitive decline.

Furthermore, to examine muscle mass and fat accumulation, samples were obtained from the quadriceps muscle in the thigh and epididymal fat. H&E staining revealed that the average cross‐sectional area of individual myofibers in the dTECM‐SLC group was significantly greater than that in the SLC group or saline group. On average, fat vacuoles in the dTECM‐SLC group were significantly smaller than those in the SLC group or saline group. The results revealed that the dTECM‐SLC group presented an increase in muscle mass and a decrease in fat accumulation (Figure 6G–J). Previous studies have linked testosterone deficiency (TD) to reduced bone mineral density (BMD), which can be alleviated by testosterone replacement therapy.^[^ ^38^ ^]^ Micro‐CT revealed that compared with SLC transplantation, dTECM‐SLC transplantation significantly increased the BMD of the femur, as well as the number of bone trabeculae in the femur (Figure 6K,L; Figure S15A–C, Supporting Information). The BMD of the tibia was also significantly greater (Figure S15D–F, Supporting Information) in the dTECM‐SLC group than in the SLC group, whereas no significant differences were observed in the number of bone trabeculae or the thickness of the bone trabeculae in the tibia (Figure S15G,H, Supporting Information). Collectively, these findings underscore the potential of dTECM‐SLC treatment to alleviate testosterone deficiency‐related symptoms in aged mice.

## 3 Conclusion 

Here, we revealed that age‐associated changes in the testis interstitium impaired the function of transplanted SLCs, causing their effectiveness to remain unsatisfactory. We developed young porcine dTECM to remodel the SLC niche. The dTECM exhibited the tissue structure and mechanics of the testicular interstitium and enhanced the growth and function of SLCs to promote LC regeneration. dTECM‐SLC treatment effectively promoted testosterone production and the restoration of male fertility in an LC‐deficient and aged mouse model. Importantly, dTECM‐SLC could also alleviate symptoms of testosterone deficiency, such as muscle loss, decreased bone density, fat accumulation, and cognitive impairment. Our preclinical studies support the application of SLC‐based therapeutic interventions for combating LC dysfunction in aged mice.

## 4 Discussion

Stem cell functions are controlled by intrinsic and extrinsic factors and are critical for tissue homeostasis and repair during aging.^[^ ^39^, ^40^ ^]^ SLCs undergo dramatic functional declines, manifested as increased abundance, clonal hematopoiesis, and LC differentiation, which could ultimately lead to testosterone deficiency. The outcomes of transplanting young SLCs in aged testes are poor, whereas the transplantation of old SLCs into the young testicular interstitium improves treatment outcomes, suggesting that the aging niche environment is hostile to transplanted cells. Studies have revealed that the damage associated with age‐related disruption of the testicular microenvironment includes increased chronic inflammation, increased oxidative stress, and immune cell infiltration.^[^ ^41^, ^42^ ^]^ All of these factors could cause the observed decline in the repair capacity of SLCs after transplantation into the testis. Notably, the stem cell niche can be recreated via biomaterial‐based scaffolds, which can be functionalized with biophysical and biochemical cues to guide stem cell fate.^[^ ^43^ ^]^ ECM‐based stem cell niche remodeling strategies have been widely used for tissue regeneration. For example, the myocardial ECM has been shown to promote the survival and differentiation of cardiac progenitor cells to promote repair and regeneration.^[^ ^44^ ^]^ In this study, we revealed that dTECM remodeled the SLC niche in vivo, which promoted SLC proliferation and differentiation into LCs. dTECM‐SLCs effectively promoted long‐term homeostasis via the replacement of endogenous LCs in LC deficiency and senescence mouse models.

Substantial research over the past few decades has established that ECM elasticity, stiffness, and source can affect cellular growth, proliferation, migration, and differentiation.^[^ ^45^ ^]^ Therefore, several factors were considered when choosing a dTECM platform for SLC niche remodeling in this study. We chose a matrix derived from young pigs because it is the most practical material for clinical use, and many porcine‐derived matrices have recently been approved by the U.S. Food and Drug Administration. During aging, the ECM undergoes constant remodeling to adapt to the microenvironment. Age‐related changes in the ECM, which can disrupt different aspects of homeostasis, may cause adverse effects. For example, young cardiac ECM was found to promote the proliferation of induced pluripotent stem cell (iPSC)‐derived cardiomyocytes, whereas aged ECM was found to accelerate the aging phenotype.^[^ ^46^ ^]^ The ECM is specific to each tissue, and that fact that proteins upregulated in young dTECM are involved in “cellular hormone metabolic processes” and “steroid hormone biosynthesis” suggests that young dTECM has the potential to provide appropriate tissue‐specific cues for SLCs, which are capable of proliferating and differentiating into LCs to produce testosterone.

SLCs are tissue‐specific mesenchymal stem cells that reside in the testicular interstitium, and SLC‐based therapies have been reported to be safe in preclinical studies on large animals. Moreover, SLCs can survive for more than one month after transplantation in aging nonhuman primate testes. Mesenchymal stem cells exhibit an immune evasion phenotype due to their low immunogenicity. Importantly, the surviving cells regulate the inflammatory properties of macrophages and create an immune microenvironment conductive to LC regeneration. In addition, the local tissue response to dTECM after injection into the testis was examined. We found no signs of chronic inflammation in the dTECM‐treated group compared with the control group, which is consistent with previous work in which decellularized porcine ECM grafts were used.^[^ ^47^ ^]^ Moreover, local injection of dTECM‐SLCs increases biomaterial retention and does not require permeability to the blood‒testis barrier,^[^ ^48^ ^]^ which enhances the effectiveness of treatment. Compared with the injection of SLCs alone, dTECM‐SLC therapy produced more effective and longer‐lasting results. This treatment was found to not only increase bone density and strength but also increase skeletal muscle mass in men with low testosterone levels. In the clinic, compared with exogenous testosterone treatment, dTECM‐SLC treatment more strongly promotes endogenous testosterone regeneration regulated by the hypothalamus, which could prevent many long‐term side effects, such as cardiovascular events and prostate cancer, reduce intratesticular testosterone production and disrupt spermatogenesis.^[^ ^49^ ^]^ However, this work has several limitations, and further investigations are needed. Obtaining large numbers of SLCs is currently a challenge in the current stage, an erobust protocol for the differentiation of iPSCs^[^ ^50^ ^]^into SLCs is need. A more detailed molecular and biophysical understanding of the morphology and components of dTECM that promote SLC function, including the key structural and signaling molecules, is critical for the future development of clinical treatments.

In summary, we demonstrated the safety and efficacy of dTECM remodeling in the aging niche through preclinical animal studies. Our findings suggest that dTECM‐SLC therapy holds promise as a potential therapeutic approach for patients with LC dysfunction.

## 5 Experimental Section

### Ethics Statement

All animal experiments described in this study were approved by the Institutional Animal Care and Use Committee of Guangzhou Huateng Biomedical Technology Co. (no. Cz03304‐2). All animal experiments are reported in accordance with the ARRIVE guidelines.

### Animals

C57BL/6J mice (aged 3 months or 24 months) were purchased from the Animal Center of the Medical Laboratory of Guangdong Province. Actinistic green mice were purchased from GuangDong GemPharmatech Co., Ltd. All experimental procedures involving animals were performed in accordance with the guidelines of the Animal Care and Use Committee of the First Affiliated Hospital of Sun Yat‐sen University (Guangzhou, China). All the mice were housed in a pathogen‐free animal facility at 20 °C and 50% humidity on a 14‐h light/10‐h dark cycle with ad libitum access to water and food. A mouse model of EDS‐induced LC dysfunction was established as follows: mice were injected intraperitoneally with a single dose of EDS (300 mg kg^−1^ body weight). Testes and serum samples from all the animals were isolated and evaluated on day 4 after treatment. Mice with significantly reduced testosterone levels were used for further experiments. At the indicated time points, the mice in each group were euthanized via an intraperitoneal injection of pentobarbital sodium solution (150 mg kg^−1^). Testes and serum from all the animals were isolated for further analysis.

### Decellularization of Pig Testis Tissue

Young porcine testis tissue was harvested from freshly slaughtered young pigs and stored at −20 °C until use. Before decellularization, the white film was removed, and the tissue was cleaned with deionized water and cut into small cubes. The testis tissue was rinsed with Triton X‐100 (0.5%) for 24 h and then washed in 1× phosphate‐buffered saline (PBS) for 24 h to remove the detergent. The tissue was subsequently rinsed with deionized water for 24 h to remove excess ions. The decellularized testes were lyophilized and cut into small pieces. dTECM stock solutions were prepared by mixing HCl (0.01 m) with pepsin (Sigma, USA; 10 mg) and dTECM (100 mg) powder and stirring vigorously for 3 days. The prepared dTECM solution was neutralized with sodium hydroxide (NaOH, 1 M) on ice to adjust the pH to neutral. Finally, the dECM was lyophilized and sterilized via γ‐irradiation (25 kGy).

### Histological Analyses of dTECM

Tissue samples were collected at random immediately after harvesting and after each cycle of decellularization as previous described^[^ ^51^ ^]^ (Diffusion‐induced phase separation 3D printed scaffolds for dynamic tissue repair). For the histological evaluation of dTECM and native tissue, H&E staining and MT staining were conducted to detect nuclear components and evaluate collagen distribution according to the manufacturer's instructions. Immunohistochemical staining was performed to determine the levels of ECM components such as collagen I, collagen IV, elastin, and laminin. Paraffin‐embedded sections were stained with an anti‐collagen I antibody (ab34710, Abcam), an anti‐collagen IV antibody (ab6586, Abcam), an anti‐elastin antibody (ab21610, Abcam), and an anti‐laminin antibody (ab11575, Abcam) according to the manufacturers’ protocols. The prepared slides were scanned with a Leica DM 750 microscope (Leica Microsystems, Mannheim, Germany).

### Scanning Tunneling Microscopy

The dTECM hydrogel was dehydrated with increasingly concentrated ethanol solutions. After lyophilization, the sample was fixed on a sample table and coated with a layer of gold and gadolinium (Denton Vacuum). Finally, images were taken with a field emission scanning electron microscope (Zeiss, Oberkochen, Germany).

### MS Analysis

MS analysis of decellularized ECM powder was performed to determine its residual protein composition and content. LC‒MS/MS was carried out at Shanghai Bioprofile Technology Co., Ltd. (China). The MS data were analyzed against the mouse database from UniProt (UniProt‐Reference proteome‐Sus scrofa (pig) [9823]‐46179‐20230427.fasta]) at https://www.uniprot.org/proteomes/UP000000589, and data interpretation and protein identification were performed. The GO terms were grouped into three categories: biological process (BP), molecular function (MF), and cellular component (CC). Proteins were considered significantly enriched in GO terms for which p < 0.05.

### Cell Isolation and Culture

Primary SLCs were isolated and cultured as follows: Briefly, testes were harvested from 7‐day‐old C57BL/6 male mice, the tunica albuginea was carefully removed, and the testes were minced into small pieces. Interstitial cells were dissociated from the seminiferous tubules via the addition of type IV collagenase (1 mg/mL) in Dulbecco's modified Eagle's medium (DMEM) at 37 °C for 15 minutes. After the addition of DMEM containing 10% fetal bovine serum (FBS, VISTECH, SE100‐011) to stop collagenase activity, the samples were centrifuged at 1200 rpm for 5 minutes at room temperature. The pellets were resuspended in PBS and filtered through a 40 µm filter. The samples were subsequently centrifuged at 1200 rpm for 5 minutes at room temperature. The CD51^+^ cells were enriched via FACS via an Influx Cell Sorter (Becton Dickinson), after which they were seeded in SLC culture medium. The medium consisted of DMEM/F12 supplemented with dexamethasone (1 nM), LIF (Sigma, LIF2005, 1 ng mL^−1^), chicken embryo extract (US Biologicals, C3999, 5%), β‐mercaptoethanol (Gibco, 21985023, 0.1 mM), N2 (Gibco, 17502001, 1%), B27 supplement (Gibco, 17504044, 2%), basic fibroblast growth factor (Invitrogen, RFGFB50, 20 ng mL^−1^), nonessential amino acids (Gibco, 11140050, 1%), epidermal growth factor (PeproTech, 31509, 20 ng mL^−1^), platelet‐derived growth factor (PeproTech, 31518, 20 ng mL^−1^), oncostatin M (PeproTech, 30010, 20 ng mL^−1^), and insulin‐transferrin‐sodium selenite (Gibco, 41400045, 1%).

### Testicular Interstitium Digestion and Cell Collection

Enzymatic dissociation of the testicular interstitium was performed as previously described.^[^ ^52^ ^]^ Briefly, the following enzymes were used for the enzymatic dissociation of testicular tissue: collagenase type I (Worthington Biochemical, LS004196, 120 U/mL), cycloheximide (CHX; Amresco, 94217, 0.1 mg/mL) and DNAse I (10104159001, Roche, 1 mg/mL DNase I) in glycerol (50%). Testes from aged mice were placed in conical tubes (15 mL) containing collagenase I/CHX (3 mL) and DNase I solution (10 µL) in DMEM/F12. The tube was shaken vigorously until the testicular tubules started to disperse and then agitated horizontally at a speed of 120 rpm for 15 min at 33 °C. The temperature and agitation speed were the same for all subsequent incubation steps. The tubules were vertically decanted for 1 min at room temperature, the supernatant was centrifuged at 1200 rpm for 5 min, and the cells were collected.

### RNA‐seq Analysis

Total RNA was isolated from cells or testes via TRIzol reagent (Fisher Scientific, 15596018) and purified via an RNeasy Mini Kit (Qiagen, Valencia, CA), and the concentration, quality, and integrity of the RNA were determined via a Qubit 2.0 fluorometer (Thermo Fisher Scientific). cDNA libraries were constructed by using a TruSeq stranded mRNA kit (Illumina) and sequenced on a NovaSeq 6000 platform (Illumina). Reads were processed with Cutadapt v1.9.1 to remove adaptors and poly‐A sequences, and the sequenced fragments were mapped to the mouse genome and assembled via CLC Main Workbench (Qiagen). The DESeq (1.30.0) package was used to calculate differential expression levels for normalized counts. Genes with counts greater than one were included in further analyses. Heatmaps of these genes were generated with the R package. Only genes whose average log‐transformed difference was greater than 1 and whose p‐adjusted value was less than 0.05 were defined as DEGs. GO analysis, including for cellular component (CC), biological process (BP), and molecular function terms, was performed with Metascape (www.Metascape.org) using the default parameters. KEGG analysis was performed to analyze the relevance of each pathway (version January 1, 2023,https://www.genome.jp/kegg/). GSEA was conducted via GSEA v4.2.3

### Flow Cytometry

The testes were minced and incubated with type IV collagenase (1 mg mL^−1^) at 37 °C for 10 minutes. DMEM supplemented with FBS (10%) was added to stop collagenase activity. The mixture was then centrifuged at 1200 rpm for 3 minutes at room temperature and filtered through a cell strainer (40 µm) into staining buffer (PBS with 0.5% bovine serum albumin (BSA)) to obtain a single‐cell suspension. Fixation and permeabilization were performed. The cells were stained with antibodies in the dark on ice at room temperature. The cells were washed with PBS, centrifuged, and diluted to a density of 1 to 5 × 10^5^ cells/mL. Afterward, the cells were washed with FACS buffer and analyzed via flow cytometry (CytoFLEX, Beckman Coulter, Krefeld, Germany). Finally, the data were processed via FlowJo software (BD Biosciences). The antibodies used were CD51‐BV421 (BD Bioscience, 740062, 1:100), anti‐LHR (Alomone labs, ALR‐010, 1:100), and donkey anti‐rabbit IgG‐PE (BioLegend, 406421, 1:100) antibodies.

### Immunofluorescence

Cells were fixed with 4% paraformaldehyde at room temperature for 20 min and washed with PBS (Fisher Scientific). For testicular samples, the testes were fixed in paraformaldehyde and embedded in paraffin after dehydration in gradient alcohol. The paraffin‐embedded tissues were sliced into 5 µm sections. Subsequently, deparaffinization and antigen retrieval were carried out. The slices were permeabilized in Triton X‐100 (0.1%) at 4 °C for 10 min and washed three times with PBS. Blocking was performed in 5% BSA (Sigma, 1933) for 1 h at room temperature. Then, the primary antibody mixture was added, and the sections were incubated at 4 °C overnight. After three washes in Triton X‐100 (0.05%) in PBS, secondary antibody diluted in PBS was added, and the samples were incubated for 1 h at room temperature. After three washes, the nuclei were stained by incubation with DAPI for 10 min. The primary antibodies used were mouse anti‐DDX4 (Abcam, ab27591, 1:500), rabbit anti‐STAR (Santa Cruz Biotechnology, sc25806, 1:100), rabbit anti‐SYCP3 (Abcam, ab15093, 1:400), rabbit anti‐α‐SMA (Abcam, ab5694, 1:500), mouse anti‐3β‐HSD (Santa Cruz Biotechnology, 1:100), rabbit anti‐CYP11A1 (Cell Signaling, 14217, 1:400), mouse anti‐Nestin (Abcam, ab134107, 1:400), and rabbit anti‐PDGFRa (Abcam, ab203491, 1:100) antibodies. The secondary antibodies used in this assay were goat anti‐mouse Alexa Fluor 488 (Invitrogen, A28175, 1:400), goat anti‐rabbit Alexa Fluor 488 (Abcam, ab150077, 1:400), and goat anti‐rabbit Alexa Fluor 555 (Abcam, ab150078, 1:400) antibodies. Confocal images were acquired via a Zeiss LSM 980 microscope with ZEN 2.3 software.

### Fertility Analysis

To evaluate the fertility of EDS‐treated mice, 12‐week‐old male mice (saline group, n = 6; SLC group, n = 6; dTECM‐SLC group, n = 6) were separately mated with 12‐week‐old female C57BL/6 mice at a ratio of 1:2. To assess the fertility of aged mice, 24‐week‐old males (saline group, n = 6; SLC group, n = 6; dTECM‐SLC group, n = 6) were separately mated with 12‐week‐old female C57BL/6 mice at a ratio of 1:2. The mating pairs were housed together for two months. The numbers of pups and litters were assessed.

### Statistics and Reproducibility

To ensure reproducibility, all H&E and immunofluorescence staining experiments were conducted with at least three biological replicates, unless mentioned otherwise. All the data are presented as the means ± SDs. All the statistical analyses were performed via GraphPad Prism 9. An unpaired two‐tailed Student's *t*‐test was used for comparisons between two groups, and one‐way ANOVA was used for comparisons among three groups. The threshold for significance was defined as a *p*‐value <0.05, and the significance of the results are indicated as follows: *p < 0.05, **p < 0.01, ***p < 0.001.

## Conflict of Interest

The authors declare no conflict of interest.

## Author Contributions

A.C. and C.Y. contributed equally to this work. X.S., A.C., C.Y., and Z.Z. designed the experiments. A.C., C.Y., and J.L. conducted the experiments. A.C. performed all the bioinformatics analyses. A.C. and C.Y. wrote the manuscript. A.C., C.Y., and J.L. helped with the data interpretation and manuscript review. X.S. supervised the project.

## Supporting information



Supporting Information

## Data Availability

The data that support the findings of this study are available from the corresponding author upon reasonable request.
