# Supplementary material for: Reconstructing the Stem Leydig Cell Niche via the Testicular Extracellular Matrix for the Treatment of Testicular Leydig Cell Dysfunction
Source: Adv Sci (Weinh). 2024 Nov 18;12(2):2410808. doi: 10.1002/advs.202410808 (PMC11727238; doi:10.1002/advs.202410808)
Supplement: Supplementary file 1 — Supporting Information [file ADVS-12-2410808-s001.docx]

**Reconstructing the stem Leydig cell niche using young testicular extracellular matrix for the treatment of testicular Leydig cell dysfunction**

*Ani Chi, Chao Yang, Jie Liu, Zhichen Zhai, Xuetao Shi**

**Experimental section**

*Oscillatory rheology*

First, 1% and 0.5% dTECM pregels were added to a 1.5 mL Eppendorf tube and placed in a 37 °C water bath for 1 h to obtain 1% and 0.5% dTECM hydrogels, respectively. The prepared 1% and 0.5% solutions were placed on a rheometer for testing. A change in the modulus of the hydrogel was observed under a constant shear strain of 1%, and another sample of the 1% or 0.5% solution was dropped onto the rheometer stage. The temperature was increased from 25 °C to 40 °C, and the change in the modulus of the dTECM was observed.

*Turbidity test*

First, a 1% or 0.5% dTECM pregel was added to a 96-well plate, avoiding bubbles. Five wells were prepared for each concentration. The plate was placed in a preheated Biotek plate (USA) at 450 nm, and the absorbance was measured every 5 minutes for 60 minutes. Through normalization, nonlinear fitting was used to determine the gelation times for the 50% and 95% gels (t0.5 and t0.95, respectively), and the gelation speed was calculated as the slope of the tangent at t0.5.

NA=(R-R_min_)/(R_max_-R_min_)

*Microstructure characterization*

The microstructure of the dTECM hydrogels was observed via scanning electron microscopy (SEM). dECM hydrogels were prepared, frozen and lyophilized. The lyophilized hydrogel was coated with 5–10 nm of gold prior to SEM.

*Quantification of the levels of DNA and ECM components*

Testis samples were collected and decellularized. Double-stranded DNA (dsDNA) was purified via a GeneJET genomic DNA purification kit (Thermo, USA), and the amount of DNA was measured via a microplate reader (BioTek, USA). The amount of total collagen was determined via a hydroxyproline assay kit (Sigma‒Aldrich, USA).

*Sperm parameter testing*

After cervical dislocation and execution, one cauda epididymis was dissected and incubated in 0.5 mL of M2 medium (Sigma, M7167) for 15 min at 37 °C to release sperm. The sperm were then loaded into assay chambers. The system automatically measured sperm movement-related parameters and the number of sperm. To calculate sperm-related parameters, the sperm dilution was set to 100.

*NOR test*

The NOR test was conducted to explore recognition memory in the aged mouse model as previously described^[1,2]^. Briefly, the mice were allowed to acclimate to an experimental apparatus (50 cm × 50 cm × 50 cm) with two identical objects. In the acquisition phase, the mice were allowed to explore the two objects freely for 10 min. In the familiarization phase, the mice were put into the same experimental apparatus with two objects for 10 min. Then, the mice were removed from the experimental apparatus for a 10 min intertrial interval, and one of the familiar objects was replaced with a novel object. Moving the nose and/or forepaws within 2 cm of the objects or touching the objects was identified as exploratory behavior. The percentage of time spent exploring the novel object relative to the total time spent exploring both objects was calculated as the discrimination index.

*Y-maze test*

Working memory and exploratory activity were measured via a Y-maze apparatus as previously described. The mice were individually placed in the central area. The Y-maze consisted of three arms. The number of entries into the arms and alterations were recorded with the EthoVisionXT video imaging system. The percentage of spontaneous alternations (%) was defined as the number of spontaneous alternations/(the total number of arm entries − 2) × 100.

*Open field test*

The open field test was used to measure anxiety-like and locomotor behavior in the mice. Aged mice in each group were placed in the center of the arena, and an overhead Basler Gig3 camera and EthoVision XT (Noldus) software were used to measure the distance traveled and the number of entries into and time spent in the central area.


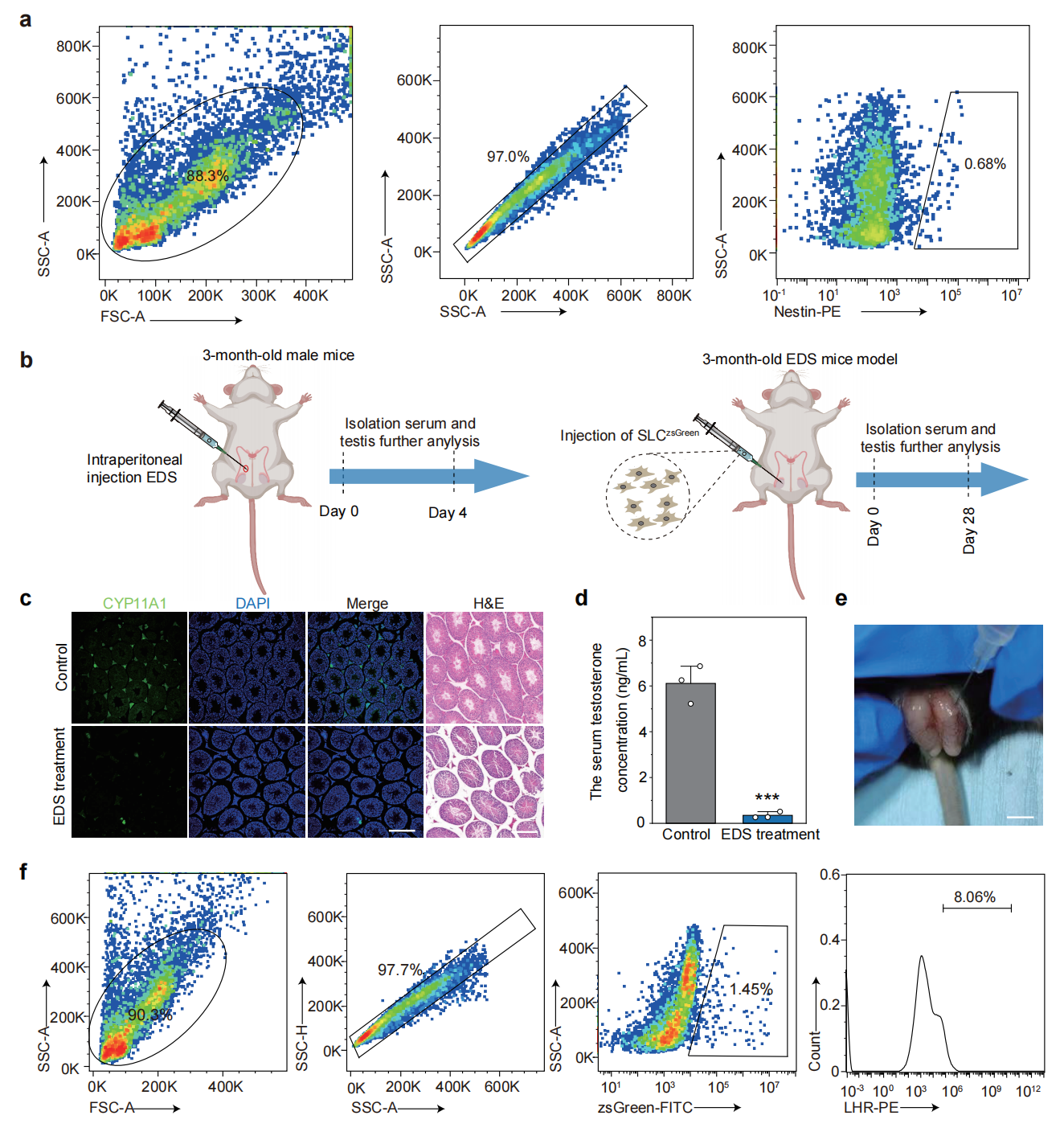
**Figure S1.** Local injection of SLCs into the testes of 3-month-old EDS-treated mice. a, Representative FACS gating scheme for SLC gating in the testes. b, Schematic illustration of SLC injection into the testicular interstitium and analysis of treatment effects in an 3-month-old EDS-treated mice. c, H&E staining (scale bar, 200 µm) and immunofluorescence staining (scale bar, 200 µm) of the LC marker CYP11A1 in EDS-treated mice on day 4. d, Quantitative analysis of serum testosterone levels in EDS-treated mice on day 4. The data are presented as the means ± SD. An unpaired two-tailed Student’s t test was performed. ****p* < 0.001, n= 3 biological replicates for each group. e, Local injection of SLCs into the testes. Scale bar, 1 cm. f, Representative FACS gating scheme for LC gating in the testes.


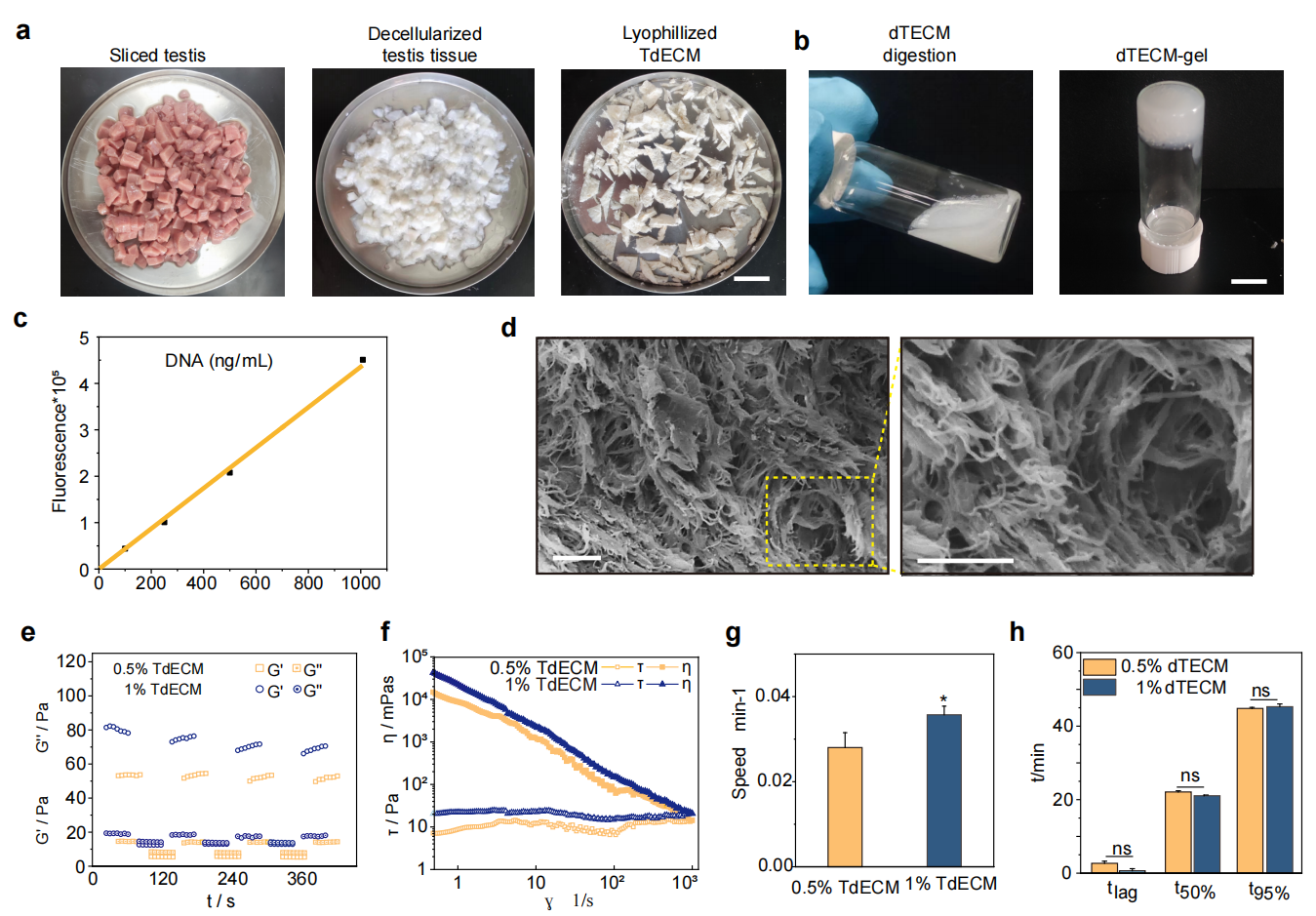
**Figure S2.** Characterization of decellularized testicular extracellular matrix and dTECM hydrogels. a, Images of decellularized extracellular matrix preparation, including chopping, washing, and freeze-drying. Scale bar, 5 cm. b, Decellularized matrix digestion and gel formation. Scale bar, 1 cm. c, Standard curve for measuring the DNA and collagen levels. d, SEM images of an ECM gel showing the fibrous collagen network. Scale bar, 1 μm; scale bar in the enlarged picture, 500 nm. e, dTECM self-healing ability. f, Shear thinning of dTECM. g, Comparison of the gelation speeds of different dTECM. h, Time for the gel to reach 50% and 95% confluence.


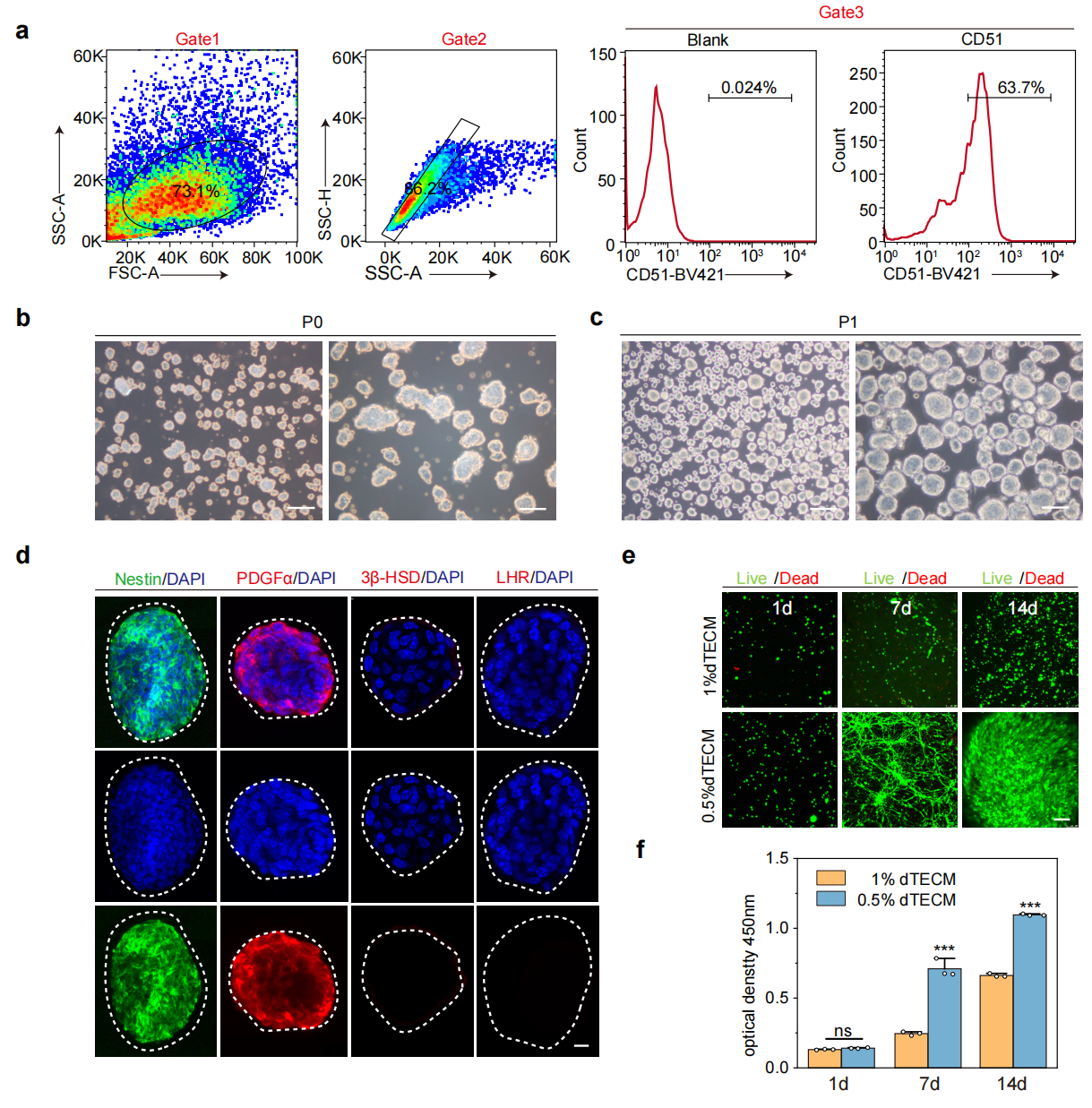
**Figure S3.** Isolation and culture of primary SLCs. a, Representative FACS gating scheme for SLC gating in the testes. b-c, Bright field image of an SLC clone. Scale bar, 100 μm; scale bar in the enlarged image, 200 μm. d, Cultured SLC spheres expressed Nestin and PDGFR-α but did not express 3β-HSD or LHR. Scale bar, 20 μm. e, Live/dead staining images of primary SLCs. Scale bar, 100 μm. f, CCK-8 cell proliferation assay. The data are presented as the means ± SDs. n= 3 biological replicates for each group. An unpaired two-tailed Student’s t test was used. ***p<0.001; ns, not significant.


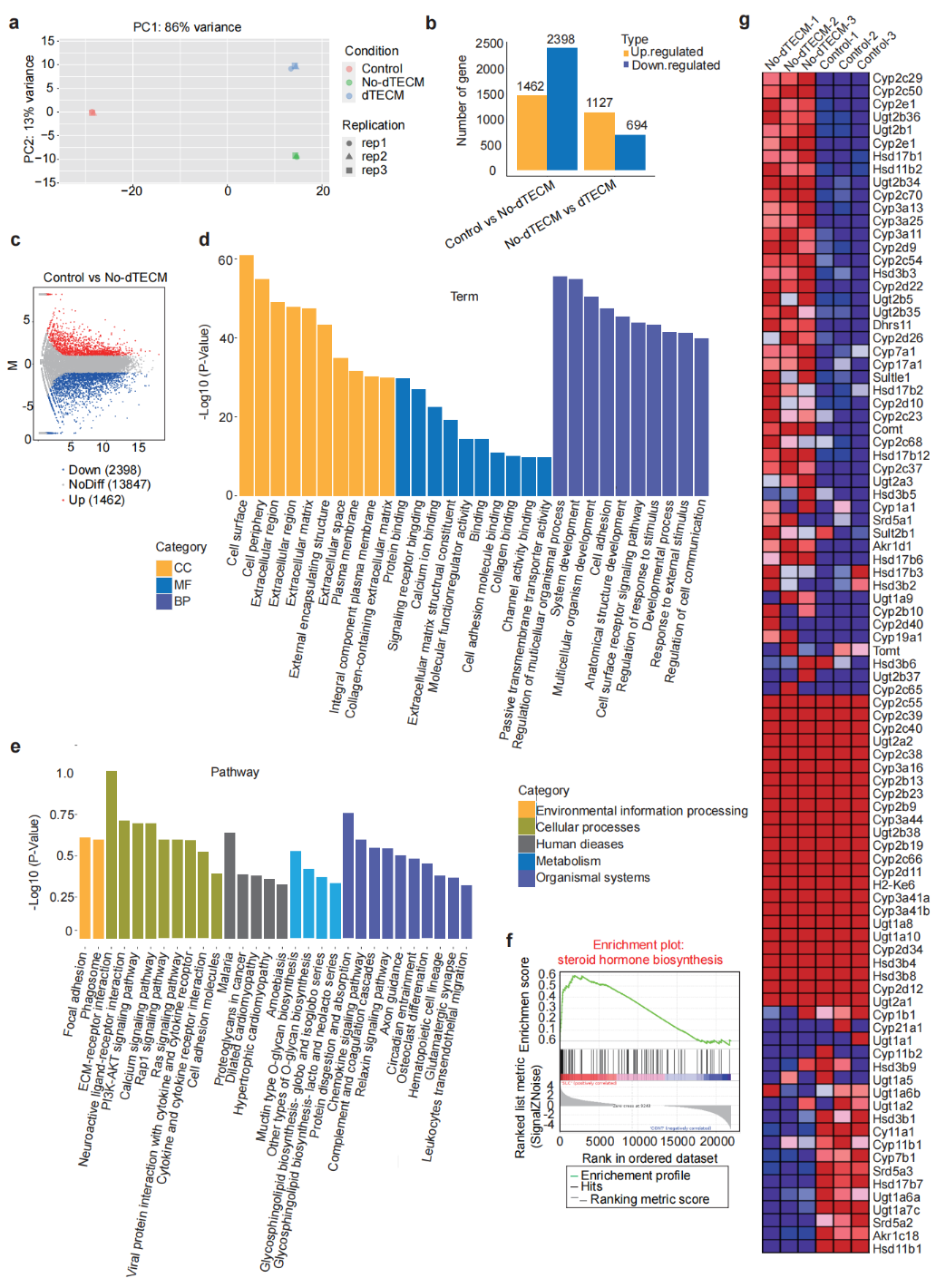


**Figure S4.** Proteins expressed in SLCs and differentiated SLCs. a, PCA of the mass spectrometry data for SLCs and differentiated SLCs (no-dTECM and dTECM groups); three biological replicates were performed for each group. b, Histogram showing downregulated and upregulated proteins in these groups. c, Volcano plot showing downregulated and upregulated proteins in SLCs and differentiated SLCs (no-dTECM group). d, GO enrichment analysis and distribution diagram of the upregulated proteins in differentiated SLCs (no-dTECM group). e, KEGG enrichment analysis and distribution diagram of the upregulated proteins in differentiated SLCs (no-dTECM group). f, GSEA showing the pathways associated with the upregulated genes in differentiated SLCs (no-dTECM group). g, Heatmap showing the expression of steroid hormone synthesis-related genes in the SLC and differentiated SLC (no-dTECM) groups.


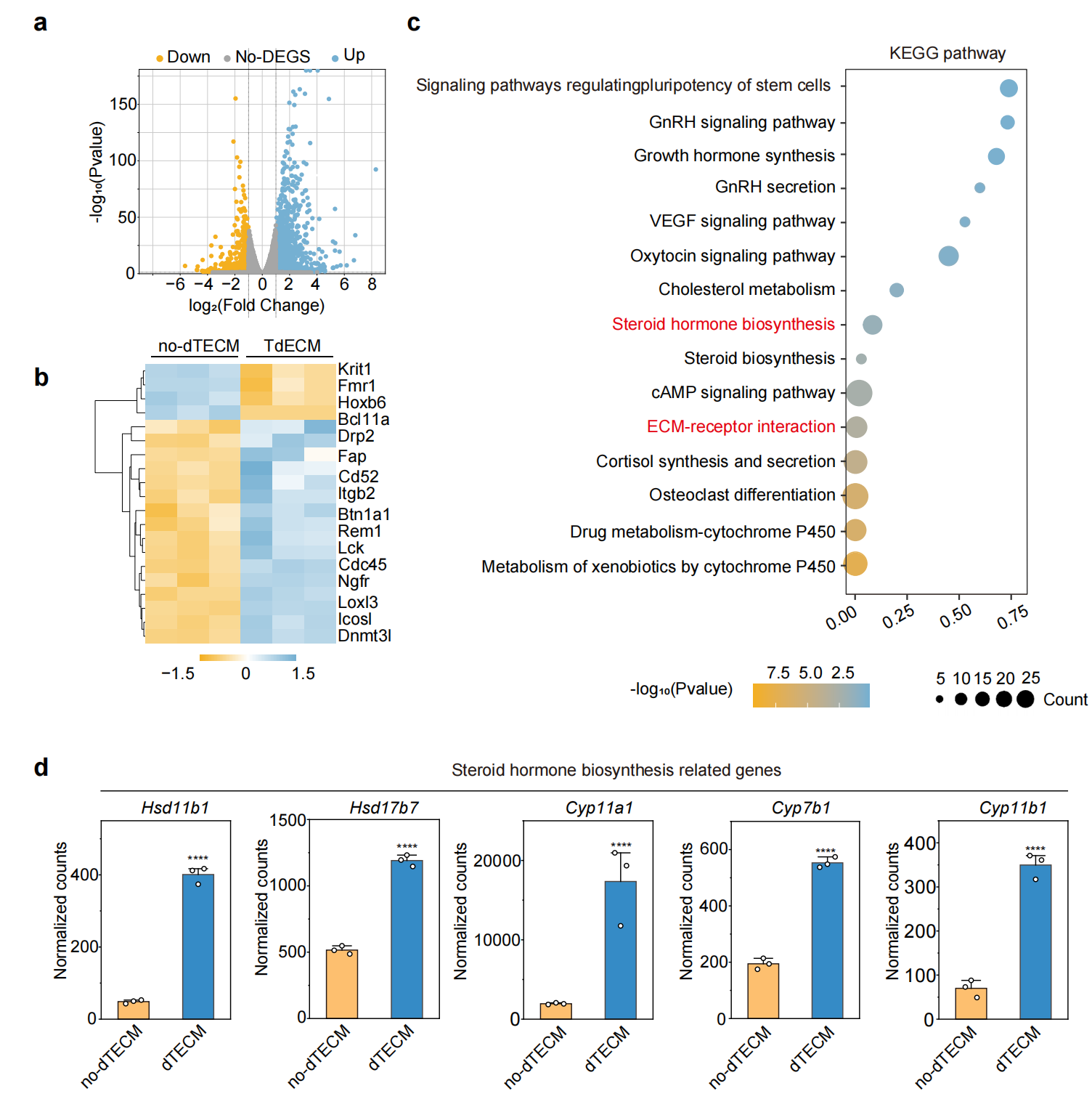
**Figure S5**. dTECM promotes SLC differentiation in vitro. a, Volcano plots of DEGs in the no-dTECM and dTECM groups. b, Heatmap of mRNA expression showing the differential expression of genes. c, Representative KEGG enrichment analysis of the upregulated genes. A hypergeometric test was used for statistical analysis. d, Bar graphs showing the differences in steroid hormone synthesis between the no-dTECM and dTECM groups (no-dTECM, n = 3 biologically independent cells; dTECM, n = 3 biologically independent cells). A hypergeometric test was used for statistical analysis. ****p<0.0001.


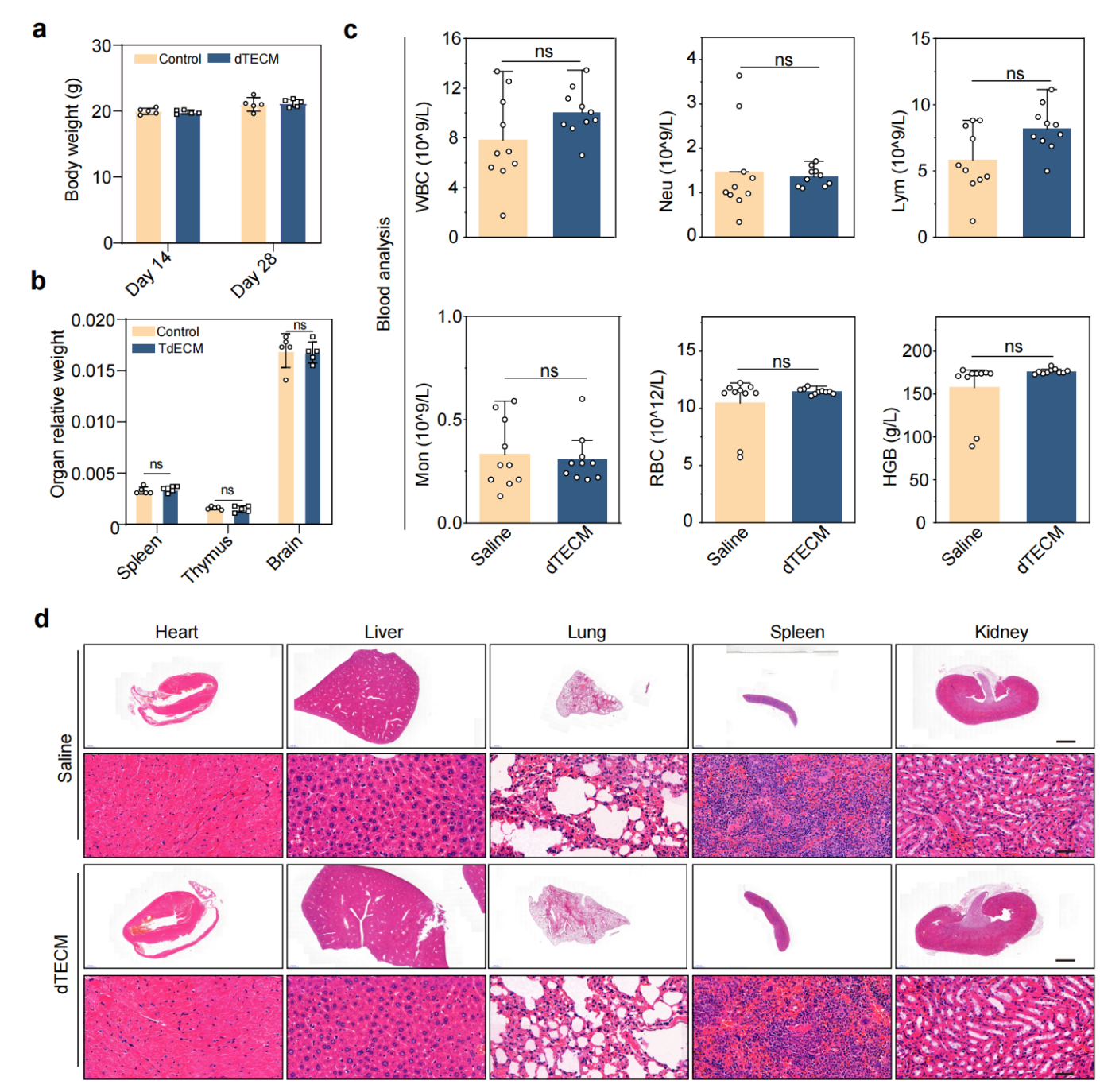
**Figure S6.** Assessment of toxicity in dTECM-treated mice. a, Quantitative analysis of the body weights of dTECM-treated mice at 14 and 28 days. The data are presented as the means ± SD. n = 5 biological replicates for each group. An unpaired two-tailed Student’s t test was performed. b, Quantitative analysis of the organ indices of control mice and dTECM-treated mice. The data are presented as the means ± SD. n = 5 biological replicates for each group. An unpaired two-tailed Student’s t test was performed. **c**, Assessment of blood biochemical indices in control mice and dTECM-treated mice at 4 weeks. Related biochemical indices included white blood cell (WBC) count, red blood cell (RBC) count, hemoglobin (HGB) level, lymphocyte (Lym) count, neutrophil count, and monocyte (Neu) count. The data are presented as the means ± SD. n = 10 biological replicates for each group. An unpaired two-tailed Student’s t test was performed. **d**, H&E staining of vital organs from control mice and dTECM-treated mice. Scale bar, 2 mm; scale bar in the enlarged picture, 25 μm. ns, not significant.


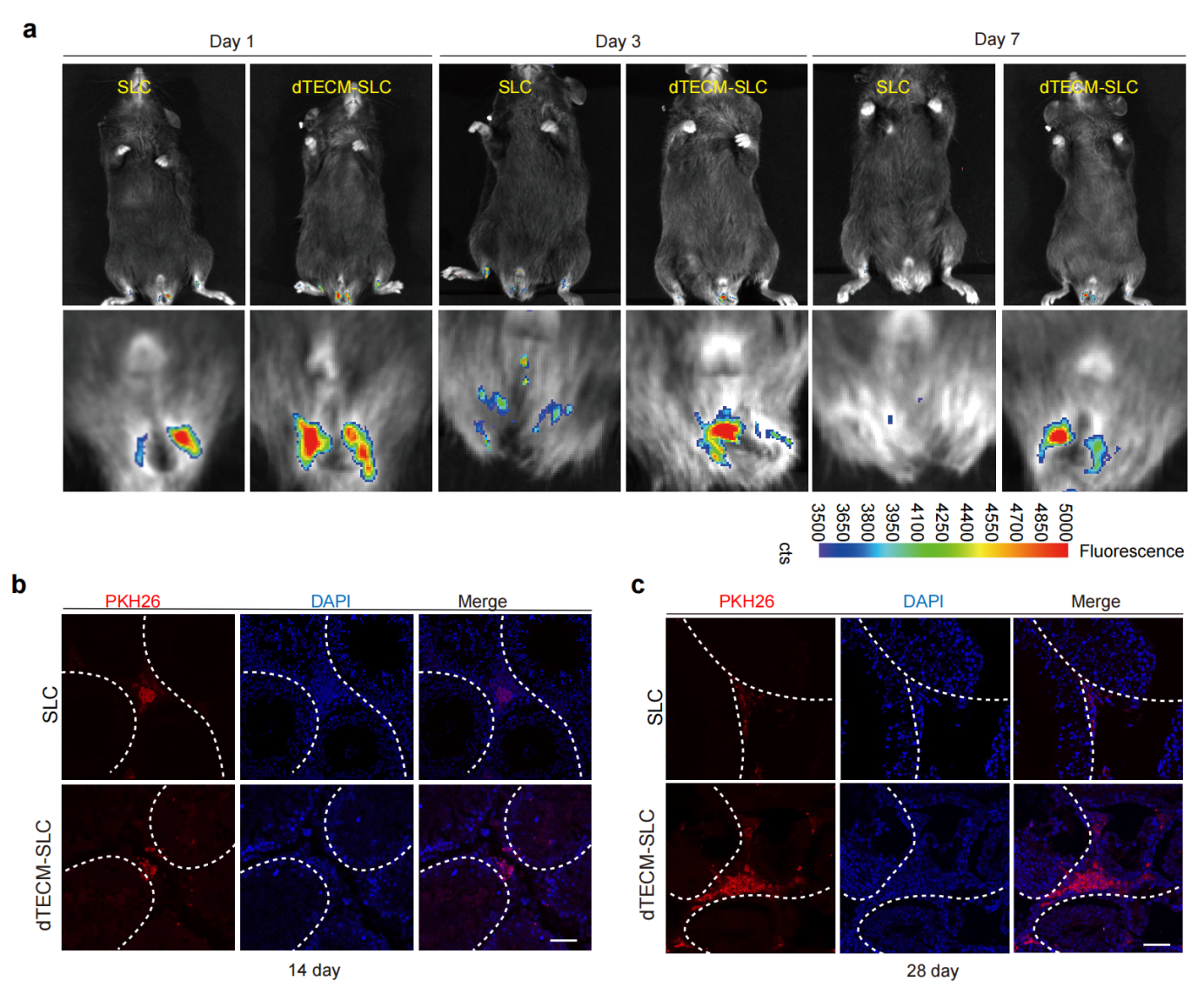
**Figure S7.** SLCs were traced after injection into the testes of EDS-treated 3-month-old mice. a, SLCs were traced with an in vivo fluorescence imaging system in the SLC and dTECM-SLC groups. b-c, Representative images of SLCs labeled with PKH26 in the testicular interstitium at 14 and 28 days in the SLC and dTECM-SLC groups. Scale bar, 50 μm.


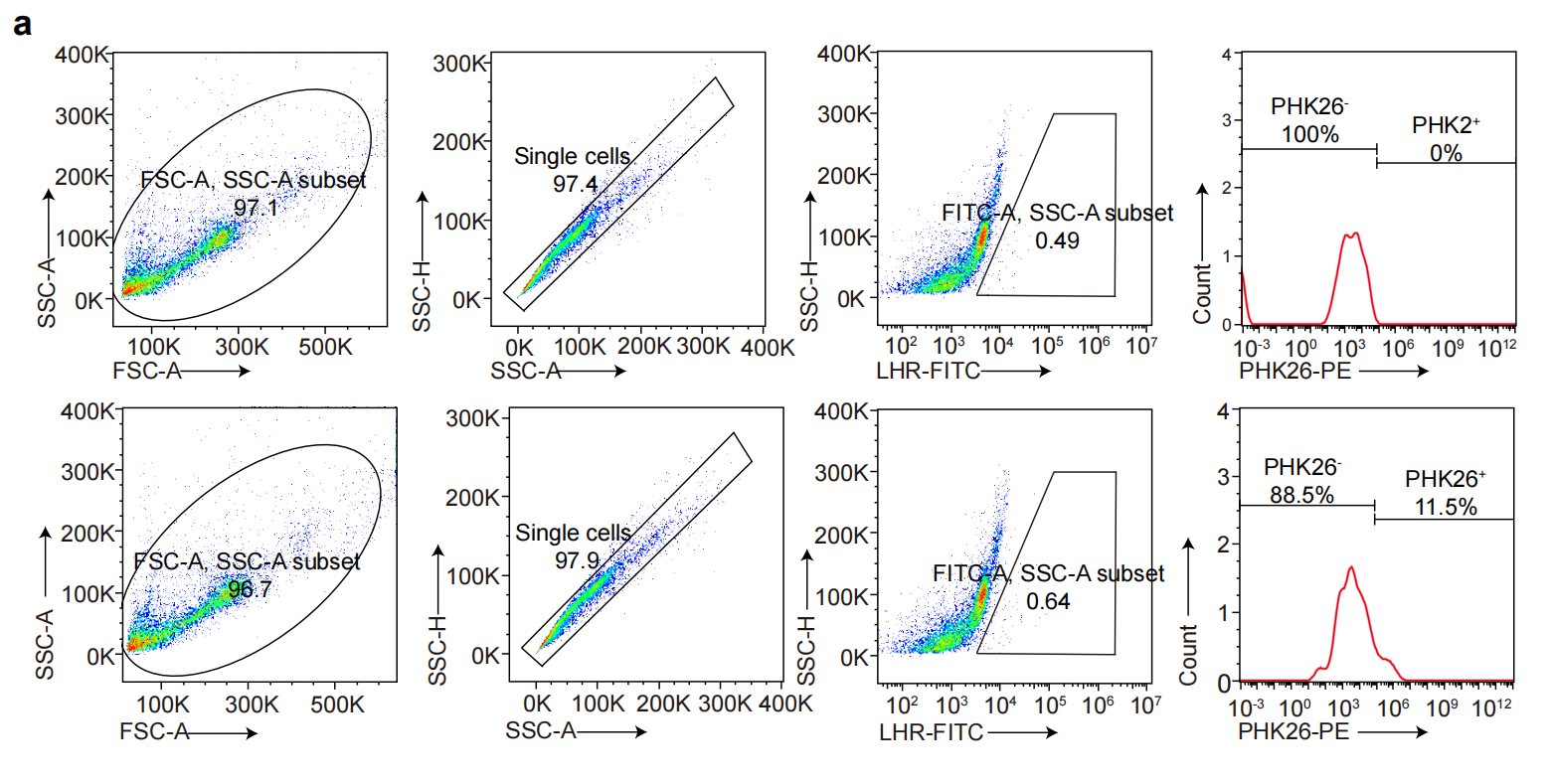
**Figure S8**. Gating strategy for flow cytometry analysis. a, Representative FACS gating scheme for LC gating in the testes.


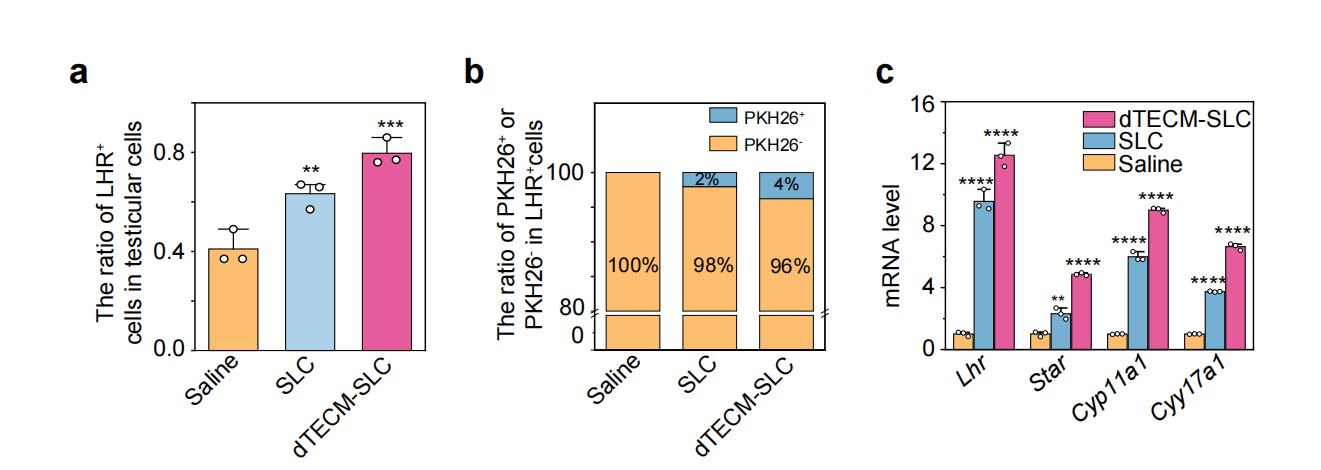
**Figure S9.** dTECM-SLC treatment promotes testosterone recovery in EDS-treated mice. a, The percentage of LHR^+^ cells was measured on day 14. n = 3 biological replicates for each group. The data are presented as the means ± SDs, and one-way ANOVA was used. b, The percentages of LHR^+^PKH26^+^ and LHR^+^PKH26^-^ cells were measured on day 14. n = 3 biological replicates for each group, and one-way ANOVA was used. c, RT‒PCR analysis of the relative mRNA expression of LC markers in testes from the saline, SLC and dTECM-SLC treatment groups on day 14. n = 3 biological replicates for each group. The data are presented as the means ± SDs, and one-way ANOVA was used. **p<0.01, ***p<0.001, ****p<0.001.


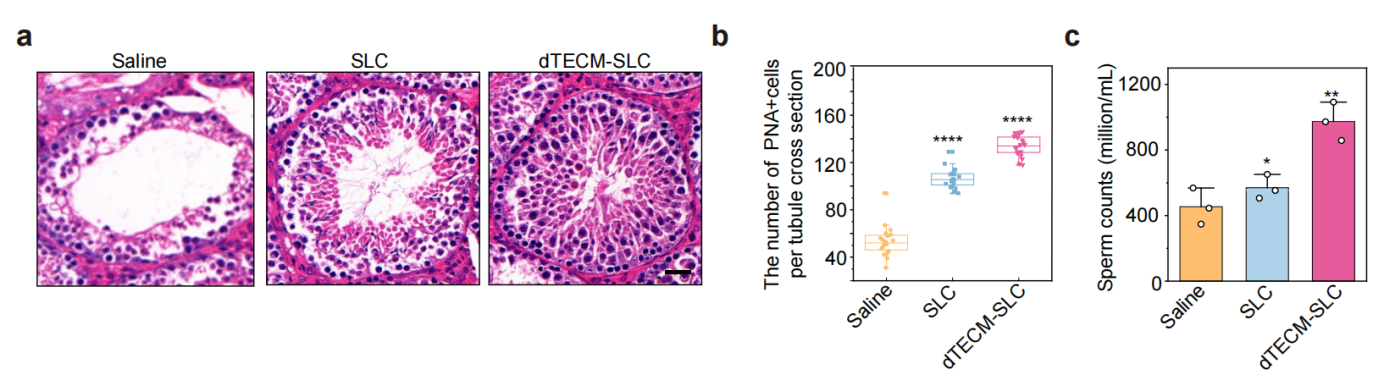
**Figure S10.** dTECM-SLC treatment promotes spermatogenesis in EDS-treated 3-month-old mice. a, H&E staining of testis samples obtained from the saline, SLC and dTECM-SLC groups on day 28. Scale bar, 20 μm. b, Sperm counts in the saline, SLC and dTECM-SLC groups. n = 3 biological replicates for each group. The data are presented as the means ± SDs, and one-way ANOVA was used. *p < 0.05, **p < 0.01, ****p < 0.0001.


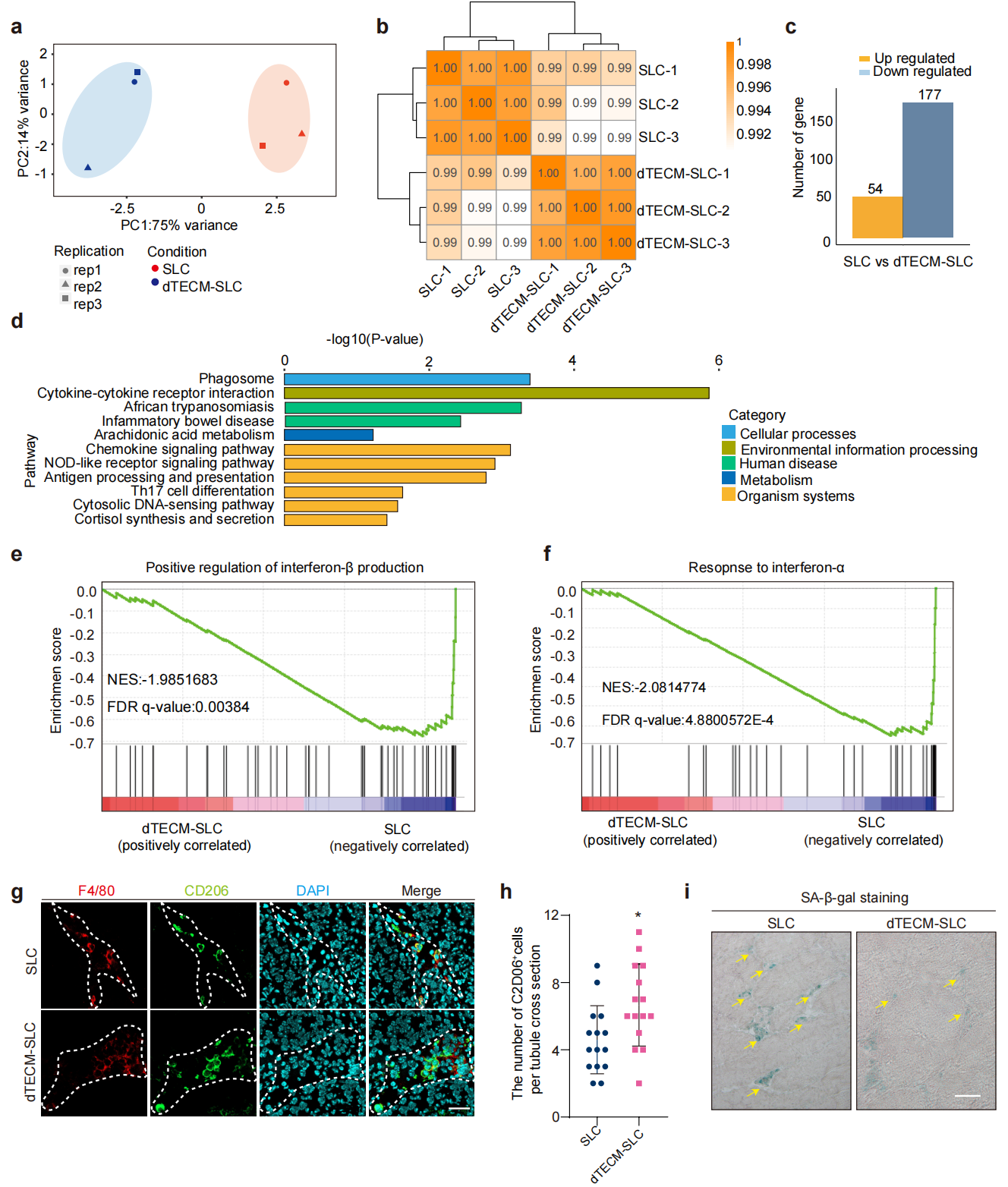
**Figure S11.** dTECM-SLC prevent chronic inflammation in an aged mouse model. a, PCA of RNA-seq data from the testes of the SLC and dTECM-SLC groups; three biological replicates were performed for each group. b, Sample correlation analysis of testes from the SLC and dTECM-SLC groups. c, Histogram showing downregulated and upregulated proteins in the SLC and dTECM-SLC groups. d, KEGG enrichment analysis and distribution diagram of the downregulated genes in the dTECM-SLC groups. A hypergeometric test was used for statistical analysis. e-f, GSEA showing the inflammation-related pathways with decreased activity in the dTECM-SLC group. g, Immunofluorescence staining of CD206 (green) in paraffin mouse testis sections. Scale bars, 25 µm. h, Quantitative analysis of the number of CD206-positive cells in paraffin mouse testis sections. n = 3 biological replicates for each group. The data are presented as the means ± SDs, and one-way ANOVA was used. i, Representative images of SA-β-gal-stained testes from aged mice treated with dTECM-SLC compared with those from mice in the SLC group. The yellow arrows point to senescent cells. Scale bars, 100 µm.


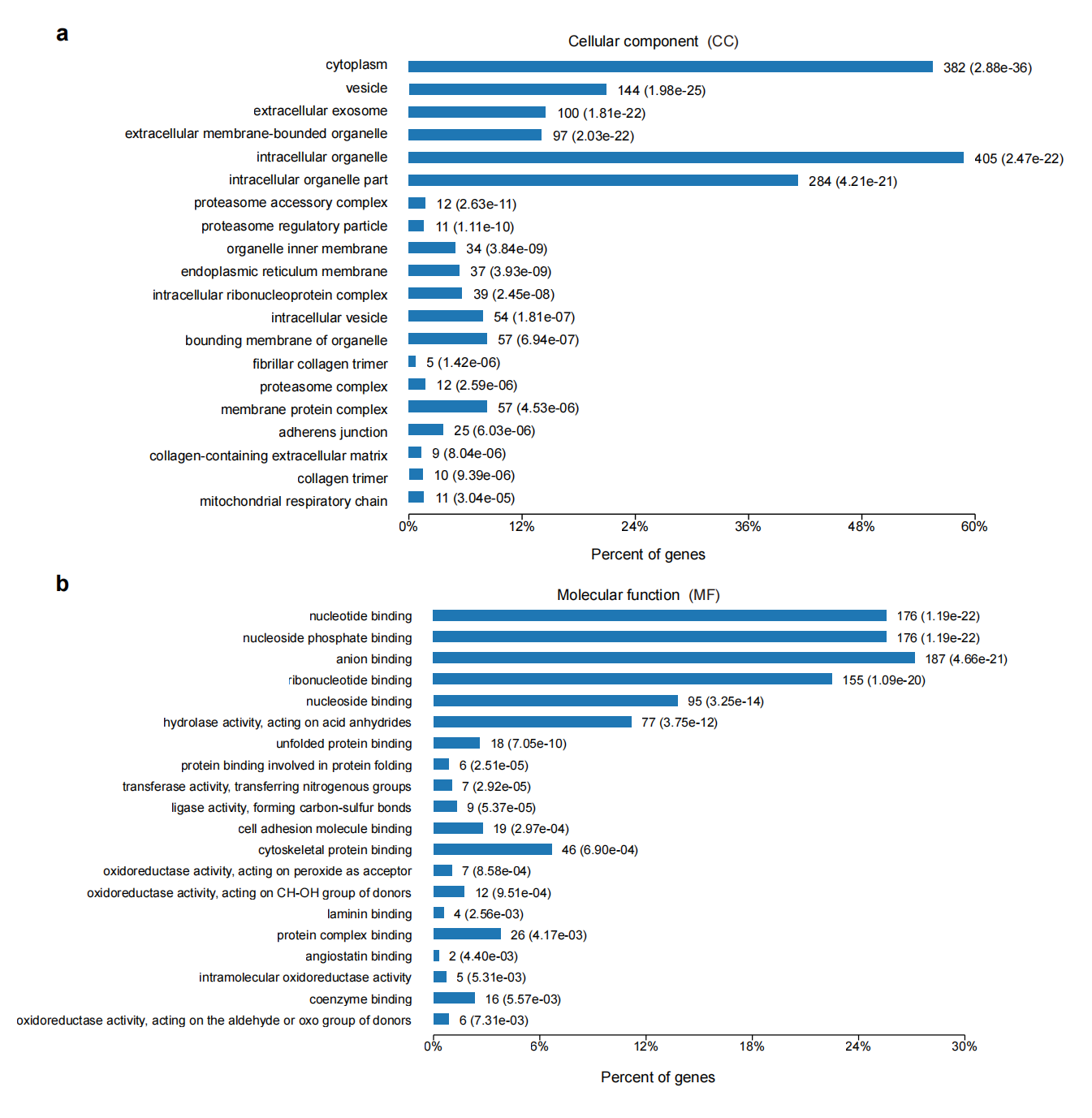
**Figure S12.** GO analysis of the proteins expressed in dTECM. a, GO CC analysis of the proteins expressed in dTECM. b, GO MF analysis of the proteins expressed in dTECM.


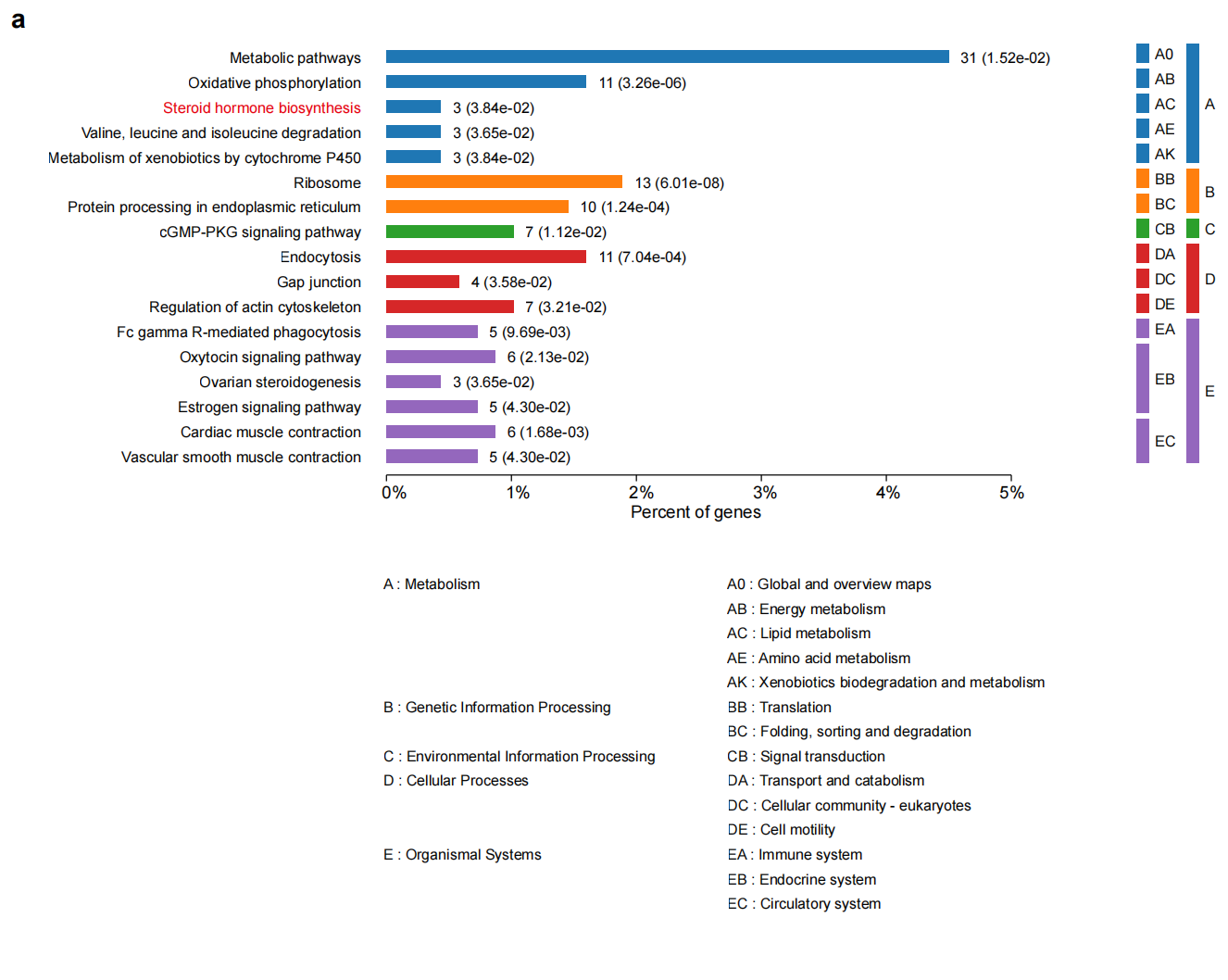
**Figure S13.** KEGG analysis of the proteins expressed in dTECM.


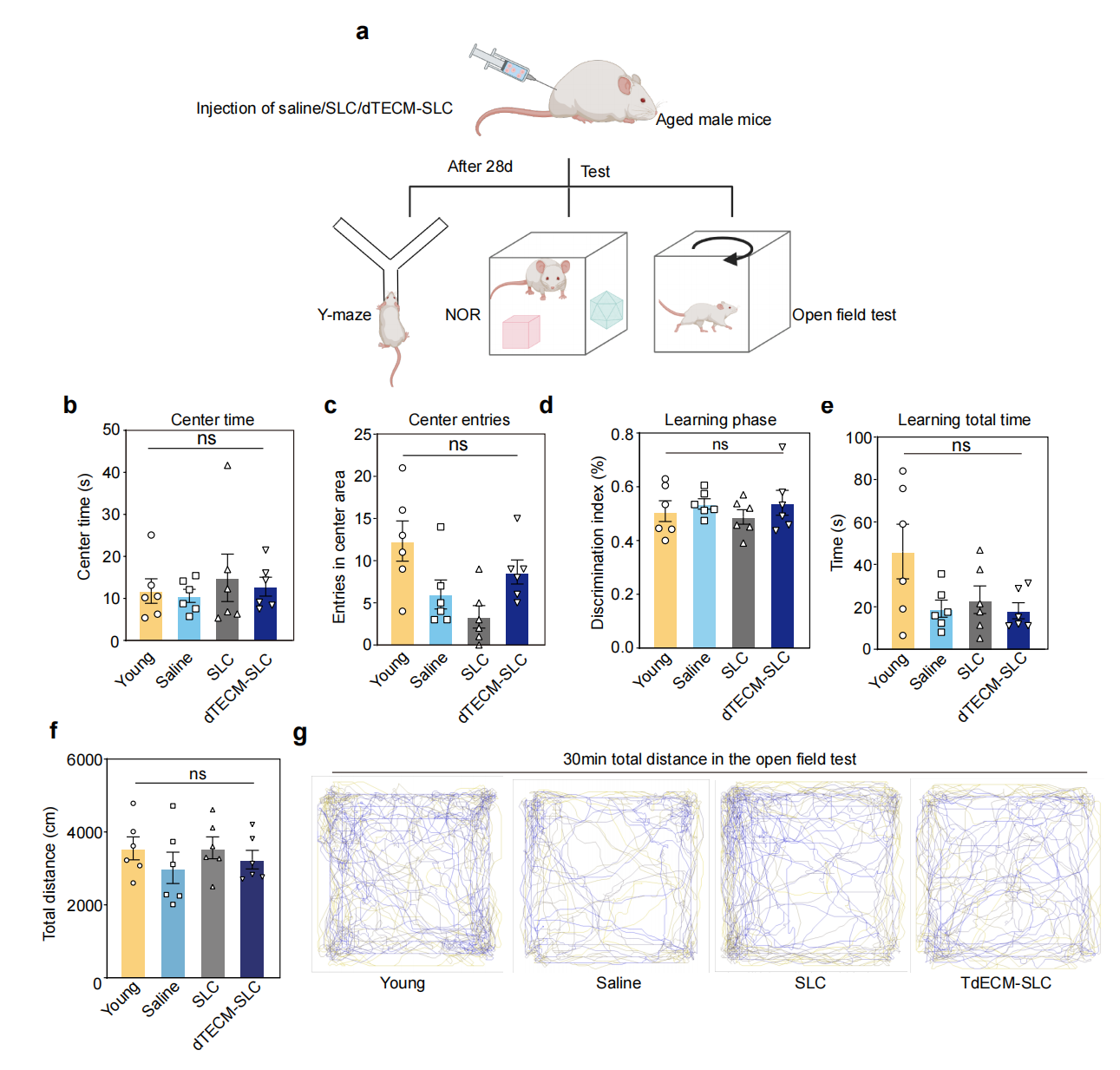
**Figure S14.** The cognitive ability of aged mice improved. a, Schematic illustration of the cognitive behavior tests. b, Time spent in the central area. n = 6 biological replicates for each group. The data are presented as the means ± SDs, and one-way ANOVA was used. c, Number of times the mouse entered the central area. n = 6 biological replicates for each group. The data are presented as the means ± SDs, and one-way ANOVA was used. d, Exploratory preference during the learning stage of the NOR test. n = 6 biological replicates for each group. The data are presented as the means ± SDs, and one-way ANOVA was used. e, Learning time during the learning stage of the NOR test. n = 6 biological replicates for each group. The data are presented as the means ± SDs, and one-way ANOVA was used. f, Total distance traveled by the mice in the open field test. n = 6 biological replicates for each group. The data are presented as the means ± SDs, and one-way ANOVA was used. g, Representative images of movement paths in the open field test. ns, not significant.


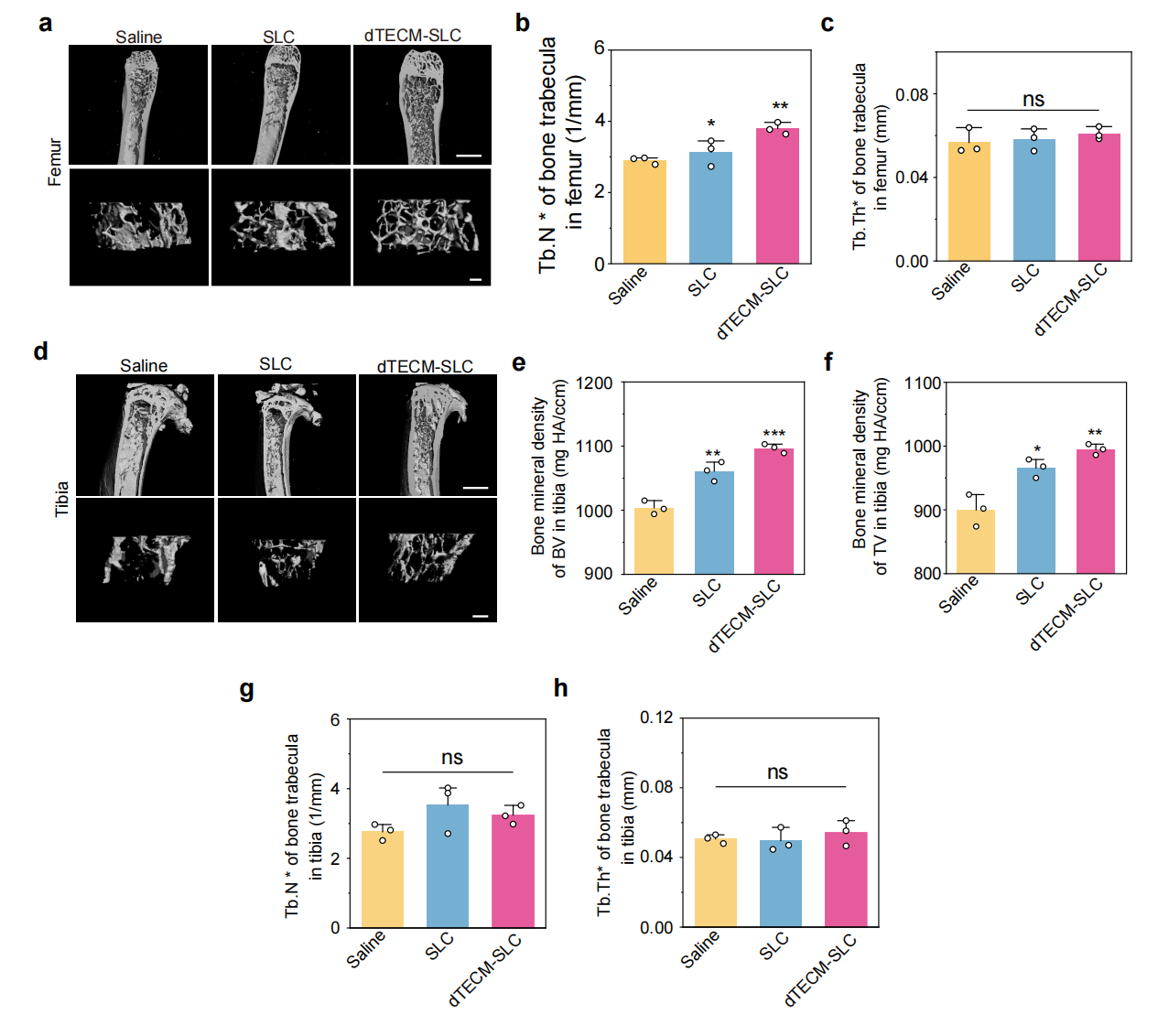
**Figure S15.** dTECM-SLC treatment alleviates osteoporosis in aging mice. a, Representative micro-CT images of the femur. The scale bar is 1 mm, and the larger scale bar is 100 μm. b-c, Quantitative analysis of the trabecular number (Tb.N) and trabecular thickness (Tb.Th) of the femur. n=3 biological replicates for each group. The data are presented as the means ± SDs. One-way ANOVA was used. d, Representative micro-CT images of the tibia. e-h, Quantitative analysis of the bone mineral density (BMD) relative to the bone volume (BV), the bone mineral density (BMD) relative to the tissue volume (TV), the trabecular number (Tb. N), and the trabecular thickness (Tb. Th) for the tibia. The scale bar is 1 mm, and the larger scale bar is 100 μm. n = 3 biological replicates for each group. The data are presented as the means ± SDs, and one-way ANOVA was used. *p < 0.05, **p<0.01, ***p<0.001. ns, not significant.

Table S1: Primers used to amplify transcripts for RT‒PCR

| Gene  Gene | Primer sequence (5′-3′) |
| --- | --- |
| *Cyp17a1* | AGTCAAAGACACCTAATGCCAAG  ACGTCTGGGGAGAAACGGT |
| *3βHsd* | AGCTCTGGACAAAGTATTCCGA  GCCTCCAATAGGTTCTGGGT |
| *Lhr* | CTCGCCCGACTATCTCTCAC  ACGACCTCATTAAGTCCCCTG |
| *Star* | CGGGTGGATGGGTCAAGTTC  GCACTTCGTCCCCGTTCTC |
| *Cyp11a1* | AGGTCCTTCAATGAGATCCCTT  TCCCTGTAAATGGGGCCATAC |
| *Gapdh* | AGGCCGGTGCTGAGTATGTC  TGCCTGCTTCACCACCTTCT |

**References**

[1]A. K. Evans, N. L. Saw, C. E. Woods, L. M. Vidano, S. E. Blumenfeld, R. K. Lam, E. K. Chu, C. Reading, M. Shamloo, Brain Behav Immun 2024, 118, 334.

[2]A. Traschutz, M. P. Kummer, S. Schwartz, M. T. Heneka, Behav Processes 2018, 157, 711.
